# Supplementary material for: Contrastive multiple correspondence analysis (cMCA): Using contrastive learning to identify latent subgroups in political parties
Source: PLoS One. 2023 Jul 10;18(7):e0287180. doi: 10.1371/journal.pone.0287180 (PMC10332614; doi:10.1371/journal.pone.0287180)
Supplement: S1 Appendix — Blackbox scaling and ordinal item response theory model. (PDF) [file pone.0287180.s001.pdf]

S1. Results of ordinary scaling: blackbox scaling and ordinal item response theory model

S1.1. CES 2020

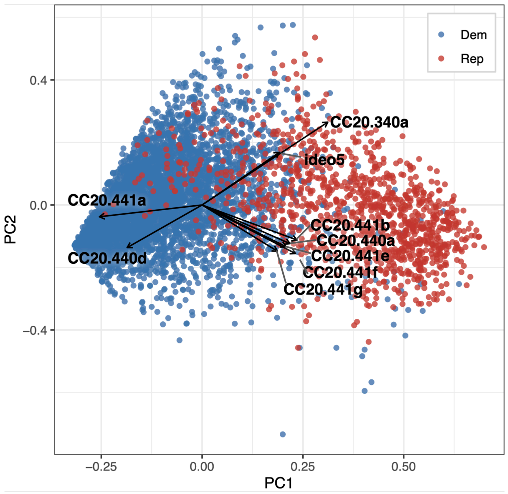

(a) BlackBox result

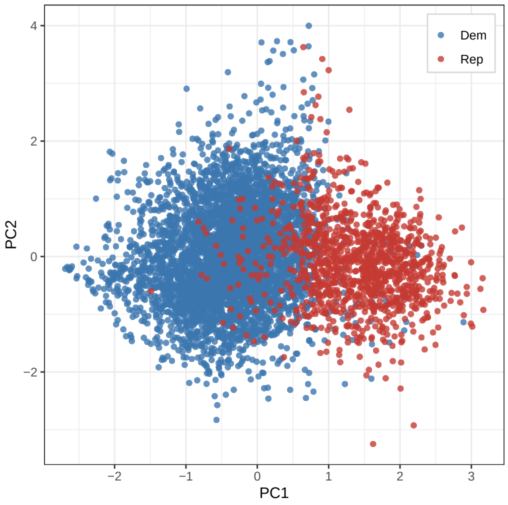

(b) OIRT result

Fig. 6: Results of CES 2020 using blackbox scaling and OIRT

We present the blackbox scaling and OIRT results of CES 2020 in Fig 6. Compared to the MCA result in Fig 1, as mentioned in the main text, although the derived plots are not identical, they all reveal one similar pattern among the U.S. voters—a high level of political polarization exists and is along with the partisan line.

S1.2. ESS 2018

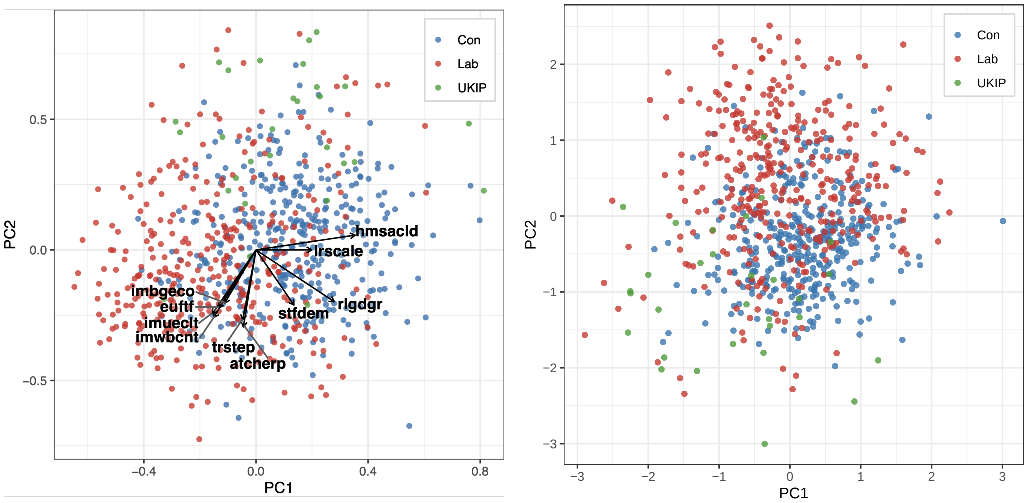

(a) BlackBox result **(b)** OIRT result

Fig. 7: Results of ESS 2018-UK using blackbox scaling and OIRT

We present the blackbox scaling and OIRT results of ESS 2018-UK in Fig 7. Compared to the MCA result in Fig 3, similar to the case of CES 2020, although the derived plots are not identical, they all reveal one similar pattern among the U.K. voters—whether polarization exists among the U.K. voters is unclear, but if it does, it is not along with the partisan line.
