# Supplementary material for: Contrastive multiple correspondence analysis (cMCA): Using contrastive learning to identify latent subgroups in political parties
Source: PLoS One. 2023 Jul 10;18(7):e0287180. doi: 10.1371/journal.pone.0287180 (PMC10332614; doi:10.1371/journal.pone.0287180)
Supplement: S2 Appendix — Category loadings and category coordinates. (PDF) [file pone.0287180.s002.pdf]

S2. Auxiliary information of MCA: category loadings and category coordinates

Here, we only list MCA's auxiliary information that is related to the first PC (PC1). For the information on the second PC (PC2), please refer to S4 appendix.

S2.1. MCA: CES 2020

From Table 1 and Fig 8, while self-identified ideology (ideo5) is not the most prominent variable comprising PC1, PC1 is consistent with the liberal-conservative ideology, as discussed in the main text.

Table 1: Variables and ranks of their value range of the categorical loadings along PC 1

CC20.320a (rank: 1)	CC20.332e (rank: 16)	CC20.340a (rank: 31)	CC20.350a (rank: 46)	CC20.334a (rank: 61)
CC20.356 (rank: 2)	CC20.330b (rank: 17)	CC20.333b (rank: 32)	CC20.334h (rank: 47)	CC20.338d (rank: 62)
CC20.350f (rank: 3)	CC20.355d (rank: 18)	CC20.330c (rank: 33)	CC20.443.2 (rank: 48)	CC20.350d (rank: 63)
CC20.350g (rank: 4)	CC20.440b (rank: 19)	ideo5 (rank: 34)	CC20.355c (rank: 49)	CC20.332b (rank: 64)
CC20.442c (rank: 5)	CC20.442b (rank: 20)	CC20.440d (rank: 35)	CC20.443.1 (rank: 50)	CC20.332f (rank: 65)
CC20.331e (rank: 6)	CC20.327a (rank: 21)	CC20.331d (rank: 36)	CC20.440c (rank: 51)	CC20.338c (rank: 66)
CC20.350c (rank: 7)	CC20.333c (rank: 22)	CC20.331b (rank: 37)	CC20.333d (rank: 52)	CC20.338b (rank: 67)
CC20.355a (rank: 8)	CC20.332d (rank: 23)	CC20.334e (rank: 38)	CC20.334f (rank: 53)	CC20.334b (rank: 68)
CC20.327d (rank: 9)	CC20.355b (rank: 24)	CC20.350e (rank: 39)	CC20.443.3 (rank: 54)	CC20.443.5 (rank: 69)
CC20.441f (rank: 10)	CC20.332a (rank: 25)	CC20.307 (rank: 40)	CC20.332c (rank: 55)	
CC20.441a (rank: 11)	CC20.331c (rank: 26)	CC20.442d (rank: 41)	CC20.355e (rank: 56)	
CC20.440a (rank: 12)	CC20.333a (rank: 27)	CC20.442a (rank: 42)	CC20.338a (rank: 57)	
CC20.441b (rank: 13)	CC20.441g (rank: 28)	CC20.334g (rank: 43)	CC20.442e (rank: 58)	
CC20.441e (rank: 14)	CC20.334c (rank: 29)	CC20.331a (rank: 44)	CC20.443.4 (rank: 59)	
CC20.350b (rank: 15)	CC20.302 (rank: 30)	CC20.334d (rank: 45)	CC20.327e (rank: 60)	

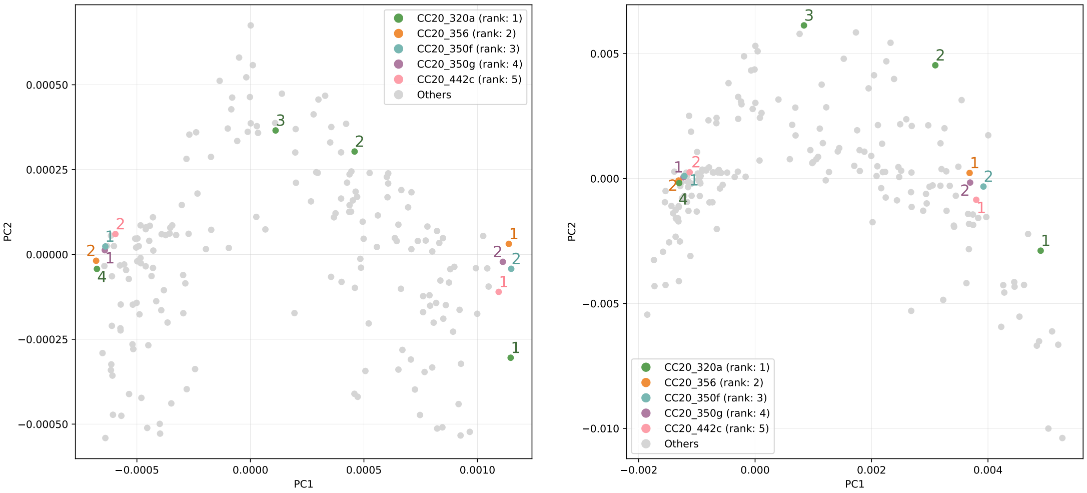

(a) Category loadings **(b)** Category coordinates

Fig. 8: Category loadings and coordinates of the top-5 variables in Table 1

S2.2. MCA: ESS 2018

From Table 2 and Fig 9, we can see the top-5 ranked variables have a clear increasing (e.g., impcntr) or decreasing (e.g., imueclt) order along PC1.

Table 2: Variables and ranks of their value range of the categorical loadings along PC 1

impcntr (rank: 1)	hmsacld (rank: 6)	freehms (rank: 11)	gincdif (rank: 16)	impenv (rank: 21)
imdfetn (rank: 2)	euftf (rank: 7)	hmsfmlsh (rank: 12)	ipstrgv (rank: 17)	stfhlth (rank: 22)
imsmetn (rank: 3)	imbgeco (rank: 8)	lrscale (rank: 13)	ipeqopt (rank: 18)	stfdem (rank: 23)
imueclt (rank: 4)	trstep (rank: 9)	imptrad (rank: 14)	ipudrst (rank: 19)	
imwbcnt (rank: 5)	atcherp (rank: 10)	trstun (rank: 15)	rlgdgr (rank: 20)	

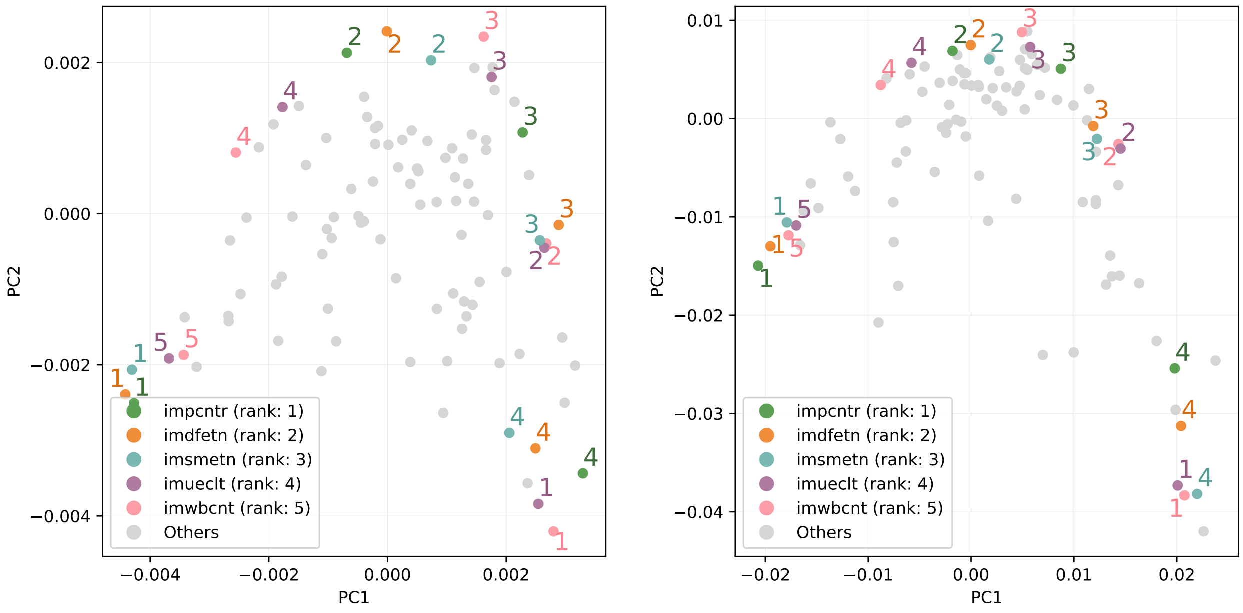

(a) Category loadings **(b)** Category coordinates

Fig. 9: Category loadings and coordinates of the top-5 variables in Table 2
