# Supplementary material for: Contrastive multiple correspondence analysis (cMCA): Using contrastive learning to identify latent subgroups in political parties
Source: PLoS One. 2023 Jul 10;18(7):e0287180. doi: 10.1371/journal.pone.0287180 (PMC10332614; doi:10.1371/journal.pone.0287180)
Supplement: S3 Appendix — Category loadings and category coordinates. (PDF) [file pone.0287180.s003.pdf]

S3. Auxiliary information of cMCA: Category loadings and category coordinates

We list cMCA's auxiliary information that is related to the first PC (cPC1). For information on the second PC (PC2) and other details, please refer to the online appendix.

S3.1. cMCA: CES 2020

From Table 3 and Fig 10, we see that the top-two issues that relate to cPC1 are: Amy Coney Barrett's confirmation (CC20.356) and Brett Kavanaugh's confirmation (CC20.350c).

Table 3: Variables and ranks of their value range of the categorical loadings along cPC1 (cMCA `Dem` vs `Rep`, CES 2020)

CC20_356 (rank: 1)	CC20_332e (rank: 16)	CC20_334b (rank: 31)	CC20_327e (rank: 46)	CC20_333c (rank: 61)
CC20_350c (rank: 2)	CC20_331b (rank: 17)	CC20_334d (rank: 32)	CC20_442e (rank: 47)	CC20_338d (rank: 62)
ideo5 (rank: 3)	CC20_440c (rank: 18)	CC20_307 (rank: 33)	CC20_441g (rank: 48)	CC20_334e (rank: 63)
CC20_441a (rank: 4)	CC20_302 (rank: 19)	CC20_320a (rank: 34)	CC20_443_5 (rank: 49)	CC20_350e (rank: 64)
CC20_340a (rank: 5)	CC20_331c (rank: 20)	CC20_355b (rank: 35)	CC20_338b (rank: 50)	CC20_350a (rank: 65)
CC20_350f (rank: 6)	CC20_331d (rank: 21)	CC20_443_3 (rank: 36)	CC20_334a (rank: 51)	CC20_333a (rank: 66)
CC20_327d (rank: 7)	CC20_442b (rank: 22)	CC20_334g (rank: 37)	CC20_333d (rank: 52)	CC20_331a (rank: 67)
CC20_350g (rank: 8)	CC20_332f (rank: 23)	CC20_332c (rank: 38)	CC20_442d (rank: 53)	CC20_350b (rank: 68)
CC20_442c (rank: 9)	CC20_355e (rank: 24)	CC20_355d (rank: 39)	CC20_334h (rank: 54)	CC20_333b (rank: 69)
CC20_443_4 (rank: 10)	CC20_440b (rank: 25)	CC20_442a (rank: 40)	CC20_334f (rank: 55)	
CC20_334c (rank: 11)	CC20_355c (rank: 26)	CC20_441f (rank: 41)	CC20_332a (rank: 56)	
CC20_332b (rank: 12)	CC20_338c (rank: 27)	CC20_441b (rank: 42)	CC20_338a (rank: 57)	
CC20_331e (rank: 13)	CC20_332d (rank: 28)	CC20_330b (rank: 43)	CC20_443_1 (rank: 58)	
CC20_440d (rank: 14)	CC20_441e (rank: 29)	CC20_330c (rank: 44)	CC20_327a (rank: 59)	
CC20_355a (rank: 15)	CC20_443_2 (rank: 30)	CC20_440a (rank: 45)	CC20_350d (rank: 60)	

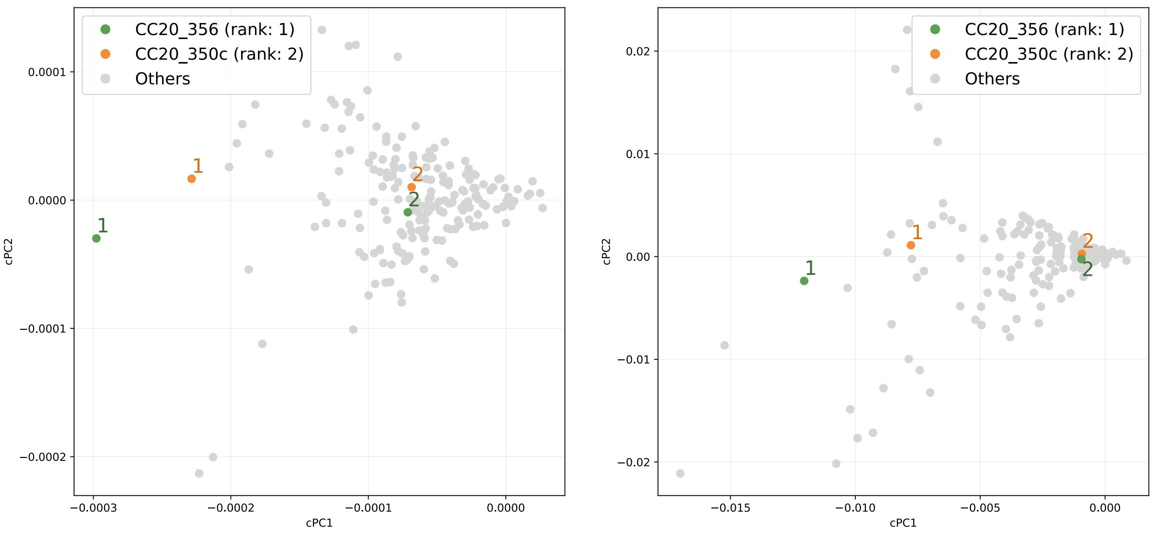

(a) Category loadings **(b)** Category coordinates

Fig. 10: Category loadings and coordinates of the variables used to subgroup `Dem` in Fig 2

From Table 4 and Fig 10, we observe that the top-two influential variables on cPC1 are all related to the approval of Trump's performance, i.e., the approve of Trump's job (`CC20.320a`) and whether to remove Trump due to abuse of power (`CC20.350f`).

Table 4: Variables and ranks of their value range of the categorical loadings along cPC1 (cMCA `Rep` vs `Dem`, CES 2020)

CC20_320a (rank: 1)	CC20_333c (rank: 16)	CC20_334e (rank: 31)	CC20_332e (rank: 46)	CC20_331d (rank: 61)
CC20_350f (rank: 2)	CC20_356 (rank: 17)	CC20_330c (rank: 32)	CC20_443_5 (rank: 47)	CC20_442e (rank: 62)
CC20_350g (rank: 3)	ideo5 (rank: 18)	CC20_307 (rank: 33)	CC20_442d (rank: 48)	CC20_334a (rank: 63)
CC20_440a (rank: 4)	CC20_442c (rank: 19)	CC20_331a (rank: 34)	CC20_331b (rank: 49)	CC20_440c (rank: 64)
CC20_441e (rank: 5)	CC20_333b (rank: 20)	CC20_355b (rank: 35)	CC20_332c (rank: 50)	CC20_338d (rank: 65)
CC20_327a (rank: 6)	CC20_327e (rank: 21)	CC20_332d (rank: 36)	CC20_443_4 (rank: 51)	CC20_338b (rank: 66)
CC20_441f (rank: 7)	CC20_332a (rank: 22)	CC20_302 (rank: 37)	CC20_334h (rank: 52)	CC20_355c (rank: 67)
CC20_350b (rank: 8)	CC20_443_2 (rank: 23)	CC20_334d (rank: 38)	CC20_334b (rank: 53)	CC20_332f (rank: 68)
CC20_340a (rank: 9)	CC20_327d (rank: 24)	CC20_443_3 (rank: 39)	CC20_338a (rank: 54)	CC20_355e (rank: 69)
CC20_355a (rank: 10)	CC20_330b (rank: 25)	CC20_334g (rank: 40)	CC20_334c (rank: 55)	
CC20_441g (rank: 11)	CC20_350c (rank: 26)	CC20_442a (rank: 41)	CC20_334f (rank: 56)	
CC20_441b (rank: 12)	CC20_350a (rank: 27)	CC20_331c (rank: 42)	CC20_338c (rank: 57)	
CC20_331e (rank: 13)	CC20_443_1 (rank: 28)	CC20_350e (rank: 43)	CC20_332b (rank: 58)	
CC20_440b (rank: 14)	CC20_442b (rank: 29)	CC20_441a (rank: 44)	CC20_440d (rank: 59)	
CC20_333a (rank: 15)	CC20_355d (rank: 30)	CC20_333d (rank: 45)	CC20_350d (rank: 60)	

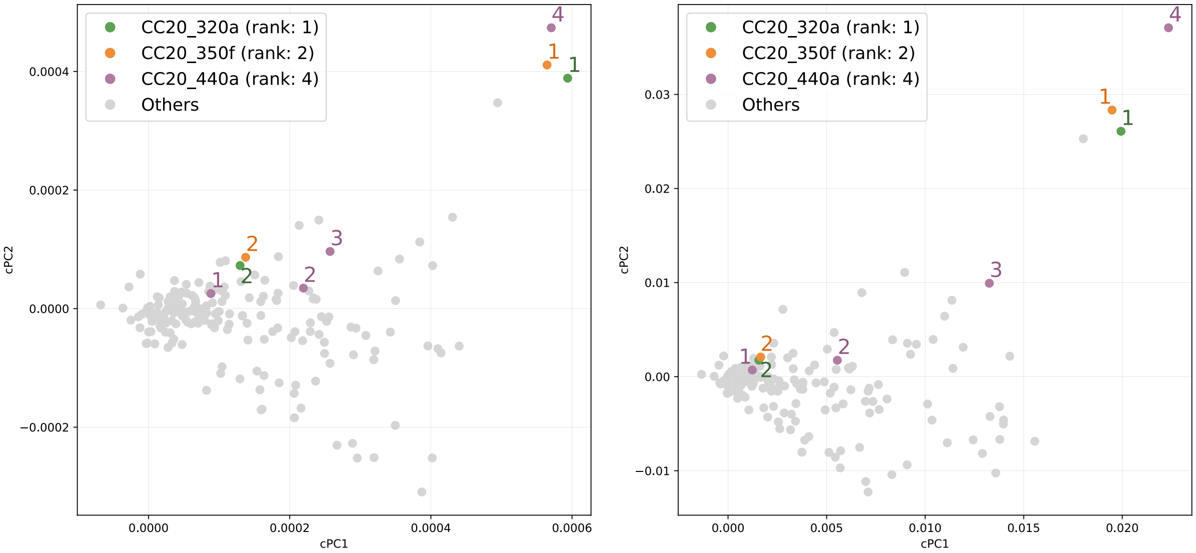

(a) Category loadings **(b)** Category coordinates

Fig. 11: Category loadings and category coordinates of the variables used to subgroup `Rep` in Fig 2

S3.2. cMCA: ESS 2018 (Fig 4)

From the below tables and figures, for both of the cMCA results, i.e., (1) target: `Lab`, background: `Con`; (2) target: `Con`, background: `Lab`, we can see that respondents' self-reported ideological position (`lrscale`) is the single most influential variable for cPC1.

Table 5: Variables and ranks of their value range of the categorical loadings along cPC1 (cMCA `Lab` vs `Con`, ESS 2018)

lrscale (rank: 1)	imueclt (rank: 6)	hmsacld (rank: 11)	ipeqopt (rank: 16)	impenv (rank: 21)
impcntr (rank: 2)	euftf (rank: 7)	hmsfmlsh (rank: 12)	atcherp (rank: 17)	trstun (rank: 22)
imdfetn (rank: 3)	imbgeco (rank: 8)	imptrad (rank: 13)	ipudrst (rank: 18)	stfhlth (rank: 23)
imsmetn (rank: 4)	freehms (rank: 9)	ipstrgv (rank: 14)	rlgdgr (rank: 19)	
imwbcnt (rank: 5)	gincdif (rank: 10)	trstep (rank: 15)	stfdem (rank: 20)	

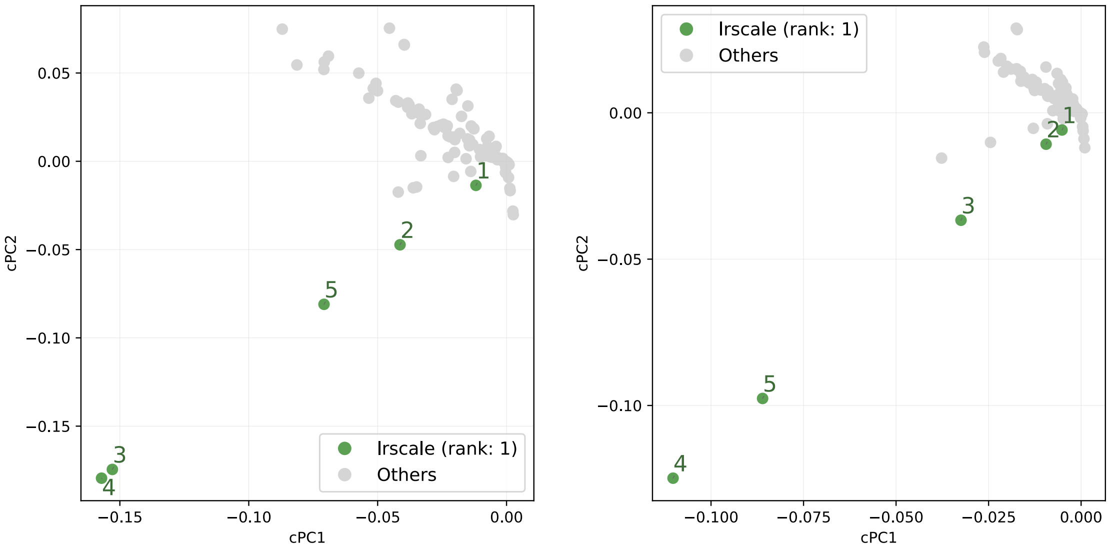

(a) Category loadings **(b)** Category coordinates

Fig. 12: Category loadings and category coordinates of the variables used to subgroup `Lab` in Fig 4

Table 6: Variables and ranks of their value range of the categorical loadings along cPC1 (cMCA `Con` vs `Lab`, ESS 2018)

lrscale (rank: 1)	imsmetn (rank: 6)	imbgeco (rank: 11)	trstep (rank: 16)	impenv (rank: 21)
gincdif (rank: 2)	freehms (rank: 7)	hmsfmlsh (rank: 12)	stfhlth (rank: 17)	rlgdgr (rank: 22)
impcntr (rank: 3)	ipeqopt (rank: 8)	imueclt (rank: 13)	ipstrgv (rank: 18)	trstun (rank: 23)
hmsacld (rank: 4)	euftf (rank: 9)	stfdem (rank: 14)	atcherp (rank: 19)	
imdfetn (rank: 5)	imwbcnt (rank: 10)	ipudrst (rank: 15)	imptrad (rank: 20)	

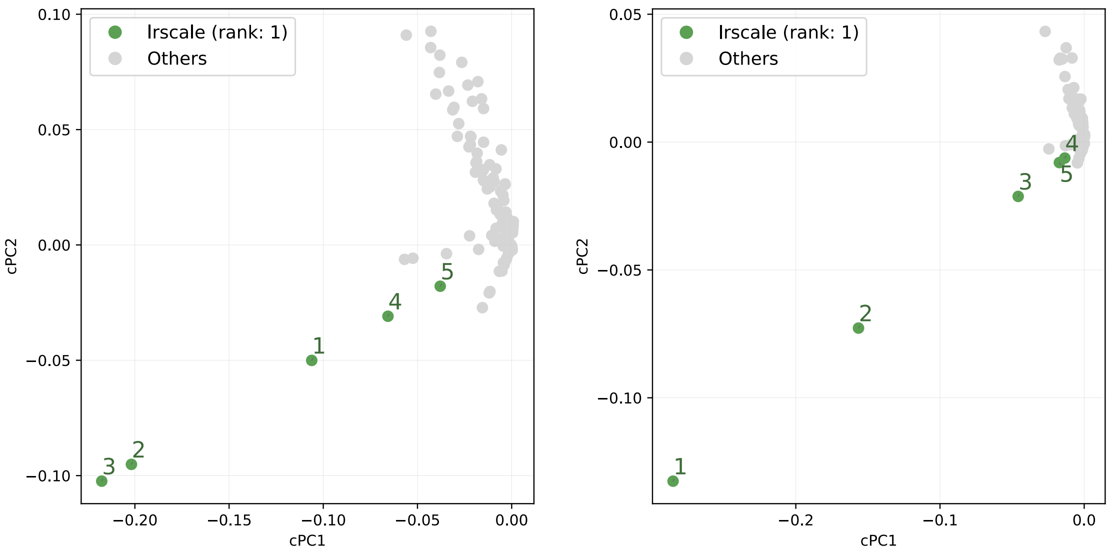

(a) Category loadings **(b)** Category coordinates

Fig. 13: Category loadings and category coordinates of the variables used to subgroup `Con` in Fig 4

S3.3. cMCA: ESS 2018 (Fig 5)

According to Table 7, the top-3 influential variables are `imwbcnt`, `imdfetn`, and `imueclt`. In addition, from Fig 14, we can see that the responses of `5 imwbcnt` (immigration makes the U.K. an *(extremely) better* place to live), the responses of `1` to `imdfetn` (allowing *many* immigrants of the different race as non-majority), and the responses of `5` to `imueclt` (the respondent's life is *extremely enriched* by immigrants) are the top-three influential categories, which "pull" some Labour supporters away from their co-partisans to the left side of cPC1. Furthermore, from the category coordinates, we infer that the respondents who hold extreme attitudes are placed to the left side (e.g., `4` or `5` to `imwbcnt`).

Table 7: Variables and ranks of their value range of the categorical loadings along cPC1 (cMCA `Lab` vs `UKIP`, ESS 2018)

imwbcnt (rank: 1)	euftf (rank: 6)	trstep (rank: 11)	rlgdgr (rank: 16)	hmsfmlsh (rank: 21)
imdfetn (rank: 2)	imsmetn (rank: 7)	stfdem (rank: 12)	gincdif (rank: 17)	imptrad (rank: 22)
imueclt (rank: 3)	atcherp (rank: 8)	trstun (rank: 13)	ipeqopt (rank: 18)	ipudrst (rank: 23)
imbgeco (rank: 4)	lrscale (rank: 9)	ipstrgv (rank: 14)	freehms (rank: 19)	
impcntr (rank: 5)	stfhlth (rank: 10)	impenv (rank: 15)	hmsacld (rank: 20)	

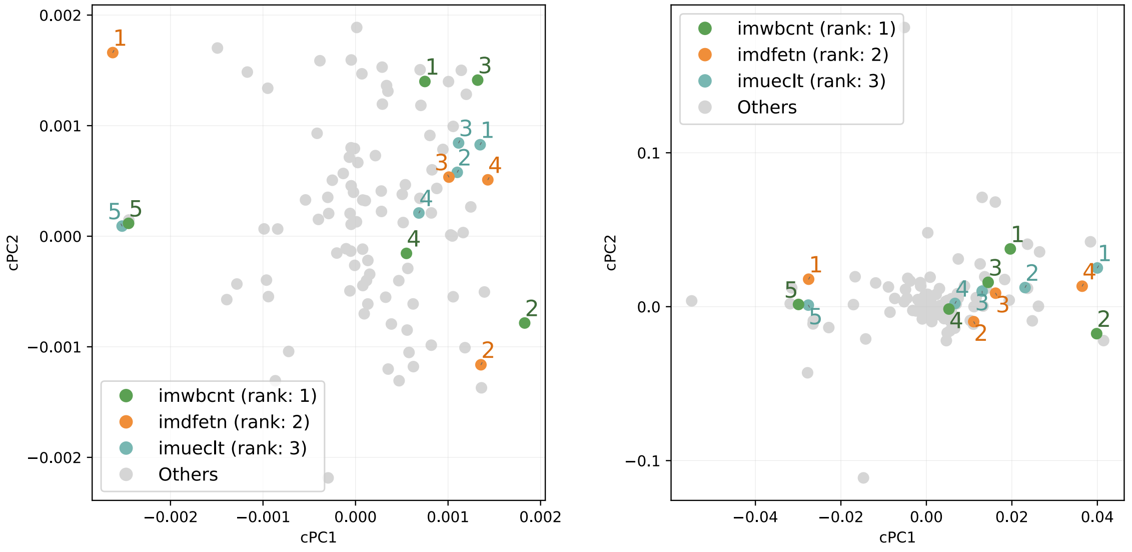

(a) Category loadings **(b)** Category coordinates

Fig. 14: Category loadings and category coordinates of the variables used to subgroup `Lab` in Fig 5

According to Table 7, the top-3 influential variables are `imwbcnt`, `imgbgeco`, and `imueclt`. In Fig 15, we see that the Conservative supporters located on the left side of cPC1 are also highly associated with the response of `5` to `imwbcnt`, `imueclt`, and `imbgeco` (the immigration makes the economy of the U.K. better).

Table 8: Variables and ranks of their value range of the categorical loadings along cPC1 (cMCA `Con` vs `UKIP`, ESS 2018)

imwbcnt (rank: 1)	trstep (rank: 6)	stfhlth (rank: 11)	freehms (rank: 16)	ipstrgv (rank: 21)
imbgeco (rank: 2)	atcherp (rank: 7)	hmsacld (rank: 12)	impenv (rank: 17)	ipudrst (rank: 22)
imueclt (rank: 3)	stfdem (rank: 8)	euftf (rank: 13)	rlgdgr (rank: 18)	imptrad (rank: 23)
trstun (rank: 4)	impcntr (rank: 9)	lrscale (rank: 14)	gincdif (rank: 19)	
imdfetn (rank: 5)	imsmetn (rank: 10)	hmsfmlsh (rank: 15)	ipeqopt (rank: 20)	

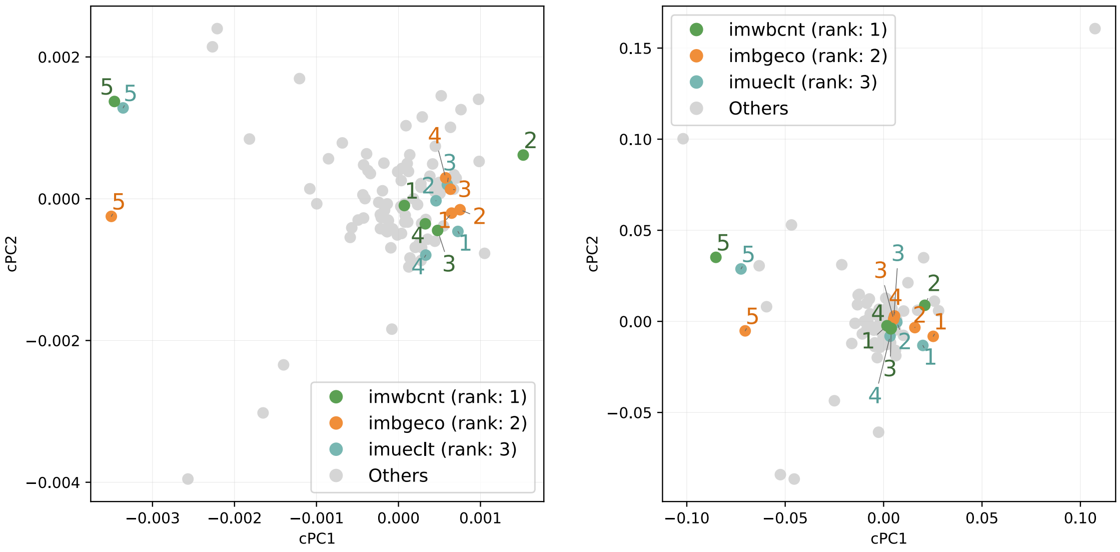

(a) Category loadings **(b)** Category coordinates

Fig. 15: Category loadings and category coordinates of the variables used to subgroup `Con` in Fig. 5
