# Supplementary material for: Contrastive multiple correspondence analysis (cMCA): Using contrastive learning to identify latent subgroups in political parties
Source: PLoS One. 2023 Jul 10;18(7):e0287180. doi: 10.1371/journal.pone.0287180 (PMC10332614; doi:10.1371/journal.pone.0287180)
Supplement: S4 Appendix — (PDF) [file pone.0287180.s004.pdf]

S4. Applying MCA to Single Groups as Comparisons

Below, we individually apply MCA to Democrats, Republicans, Labours, and Conservatives and provide data-point coordinates and category loadings of PC1 of each result.

S4.1. Democrats and Republicans

When only looking at the data-point coordinates (Fig 16a and b), we cannot see strongly distinct pattern differences from Fig. 2a and b (as mentioned in **Introduction**). However, the category loadings (Fig 16c and d) show the differences in the top-5 most influential variables derived by MCA and cMCA.

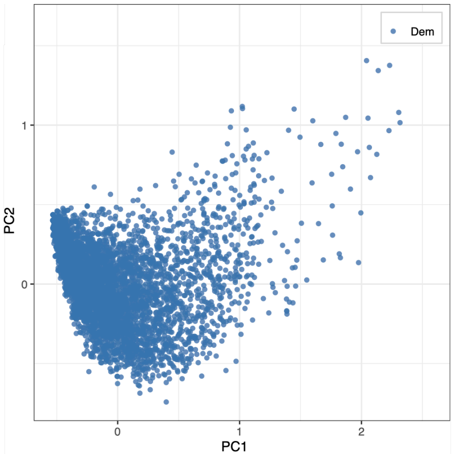

(a) Data-point coordinates of Democrats only

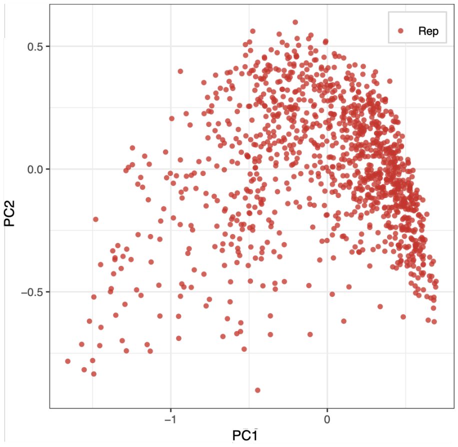

(b) Data-point coordinates of Republicans only

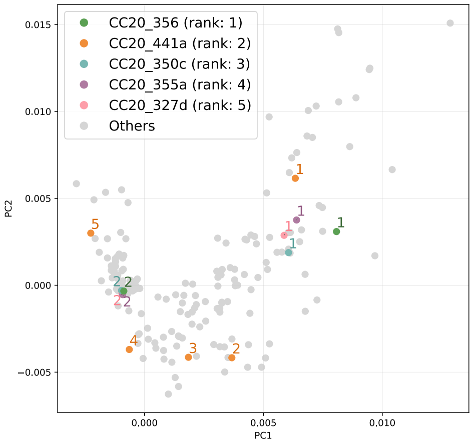

(c) Category loadings (PC1) of Democrats only

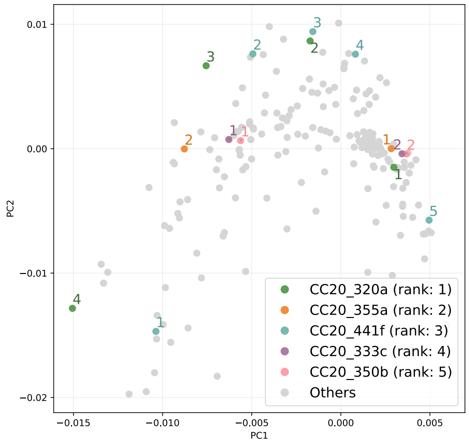

(d) Category loadings (PC1) of Republicans only

Fig. 16: MCA results of Democrats and Republicans only

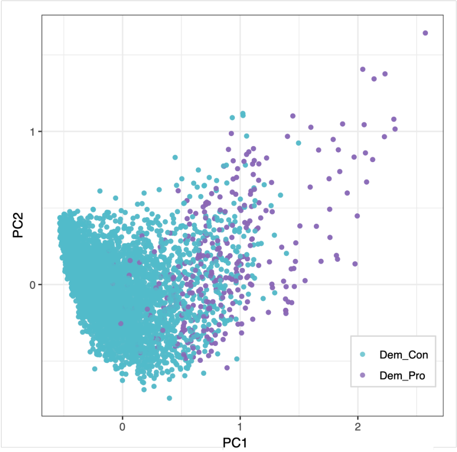

(a) Democrats only (color-coded by the attitude to the Supreme Court judge nominations)

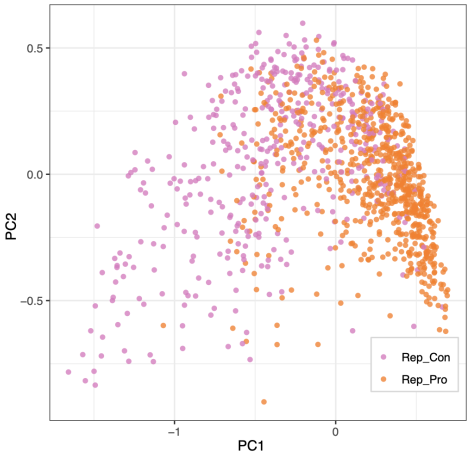

(b) Republicans only (colored by the attitude to Donald Trump's performance)

Fig. 17: Data-point coordinates of Democrats and Republicans only (color-coded by the same schema as Fig 2c and d)
