# Supplementary material for: Contrastive multiple correspondence analysis (cMCA): Using contrastive learning to identify latent subgroups in political parties
Source: PLoS One. 2023 Jul 10;18(7):e0287180. doi: 10.1371/journal.pone.0287180 (PMC10332614; doi:10.1371/journal.pone.0287180)
Supplement: S5 Appendix — (PDF) [file pone.0287180.s005.pdf]

S5. Auxiliary information of MCA and cMCA in detail

S5.1. MCA's category loadings and category coordinates (PC2): CES 2020

Table 10: Variables and ranks of their value range of the categorical loadings along PC2 (MCA, CES 2020)

CC20.440a (rank: 1)	CC20.307 (rank: 16)	CC20.338d (rank: 31)	CC20.333d (rank: 46)	CC20.331d (rank: 61)
CC20.441f (rank: 2)	CC20.320a (rank: 17)	CC20.338a (rank: 32)	CC20.355a (rank: 47)	CC20.327d (rank: 62)
CC20.441e (rank: 3)	CC20.302 (rank: 18)	CC20.350e (rank: 33)	CC20.442b (rank: 48)	CC20.355d (rank: 63)
CC20.440b (rank: 4)	CC20.443.5 (rank: 19)	CC20.350b (rank: 34)	CC20.333c (rank: 49)	CC20.332f (rank: 64)
CC20.441a (rank: 5)	CC20.355e (rank: 20)	CC20.330b (rank: 35)	CC20.355b (rank: 50)	CC20.332a (rank: 65)
CC20.441b (rank: 6)	CC20.334d (rank: 21)	CC20.333b (rank: 36)	CC20.332d (rank: 51)	CC20.356 (rank: 66)
ideo5 (rank: 7)	CC20.332b (rank: 22)	CC20.334f (rank: 37)	CC20.334b (rank: 52)	CC20.350g (rank: 67)
CC20.340a (rank: 8)	CC20.331b (rank: 23)	CC20.334e (rank: 38)	CC20.350c (rank: 53)	CC20.442d (rank: 68)
CC20.441g (rank: 9)	CC20.350d (rank: 24)	CC20.334h (rank: 39)	CC20.442e (rank: 54)	CC20.327e (rank: 69)
CC20.443.4 (rank: 10)	CC20.332c (rank: 25)	CC20.330c (rank: 40)	CC20.355c (rank: 55)	
CC20.440d (rank: 11)	CC20.334g (rank: 26)	CC20.338c (rank: 41)	CC20.332e (rank: 56)	
CC20.443.1 (rank: 12)	CC20.333a (rank: 27)	CC20.331a (rank: 42)	CC20.331c (rank: 57)	
CC20.443.2 (rank: 13)	CC20.334c (rank: 28)	CC20.334a (rank: 43)	CC20.327a (rank: 58)	
CC20.440c (rank: 14)	CC20.338b (rank: 29)	CC20.442c (rank: 44)	CC20.331e (rank: 59)	
CC20.443.3 (rank: 15)	CC20.350a (rank: 30)	CC20.442a (rank: 45)	CC20.350f (rank: 60)	

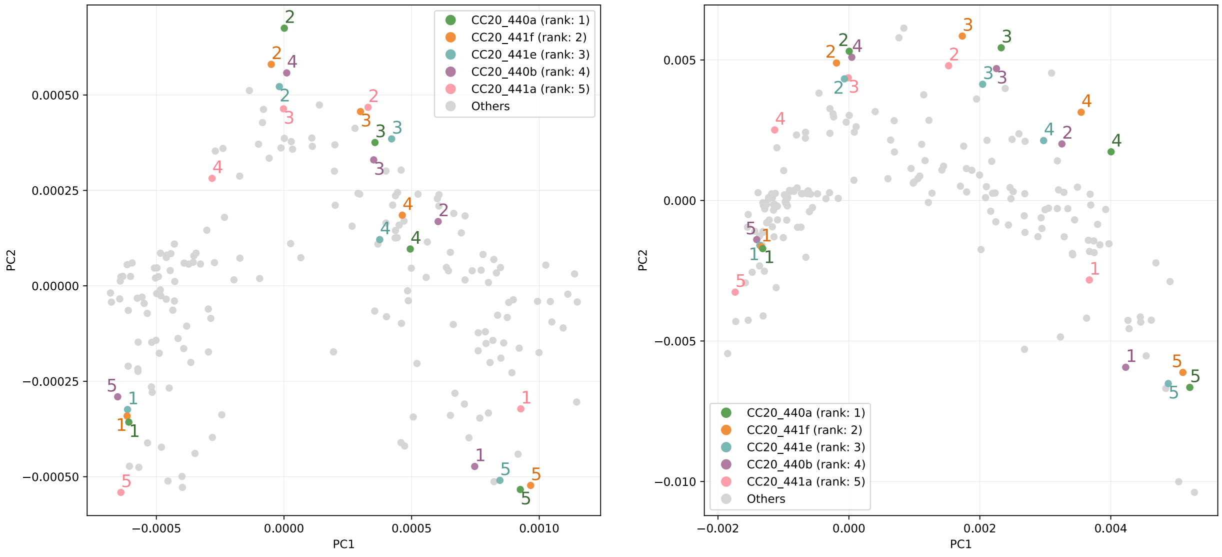

(a) Category loadings **(b)** Category coordinates

Fig. 16: Category loadings and coordinates of the top-5 variables in Table 10 (MCA, CES 2020)

S5.2. MCA's category loadings and category coordinates (PC2): ESS 2018

Table 11: Variables and ranks of their value range of the categorical loadings along PC2 (MCA, ESS 2018)

imwbcnt (rank: 1)	imsmetn (rank: 6)	trstep (rank: 11)	trstun (rank: 16)	ipudrst (rank: 21)
imueclt (rank: 2)	stfdem (rank: 7)	freehms (rank: 12)	euftf (rank: 17)	impenv (rank: 22)
impcntr (rank: 3)	lrscale (rank: 8)	hmsfmlsh (rank: 13)	imptrad (rank: 18)	ipeqopt (rank: 23)
imdfetn (rank: 4)	atcherp (rank: 9)	stfhlth (rank: 14)	rlgdgr (rank: 19)	
imbgeco (rank: 5)	gincdif (rank: 10)	hmsacld (rank: 15)	ipstrgv (rank: 20)	

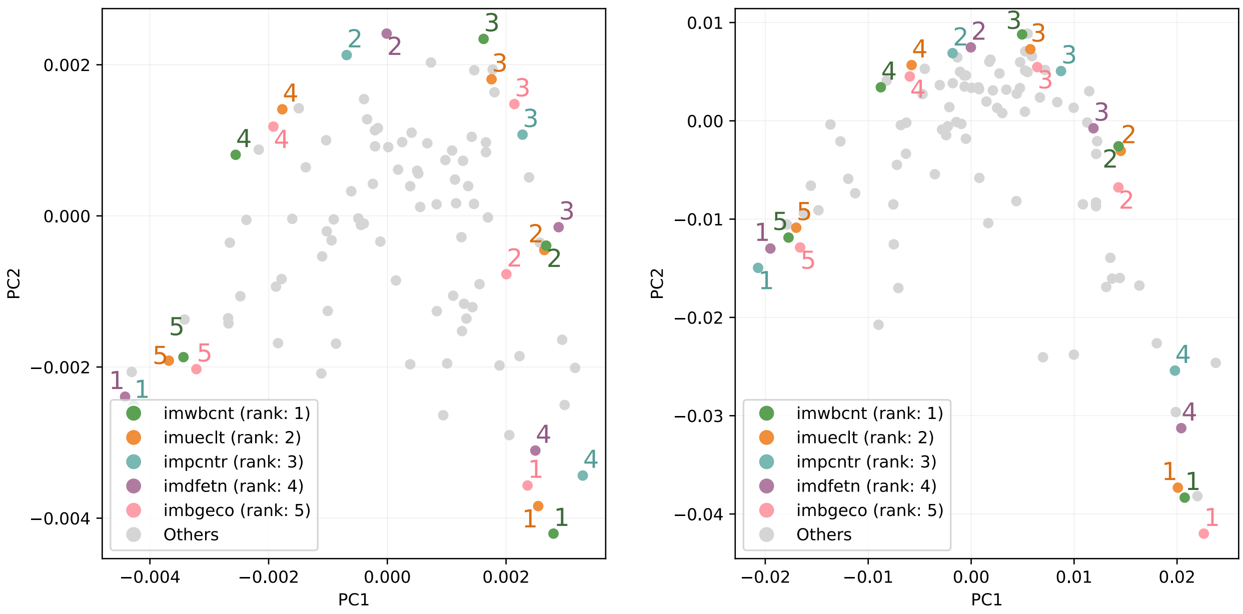

(a) Category loadings

(b) Category coordinates

Fig. 17: Category loadings and coordinates of the top-5 variables in Table 11 (MCA, ESS 2018)

S5.3. cMCA's category loadings and coordinates of the top-5 variables (cPC1): CES 2020 (target: `Dem`, background: `Rep`)

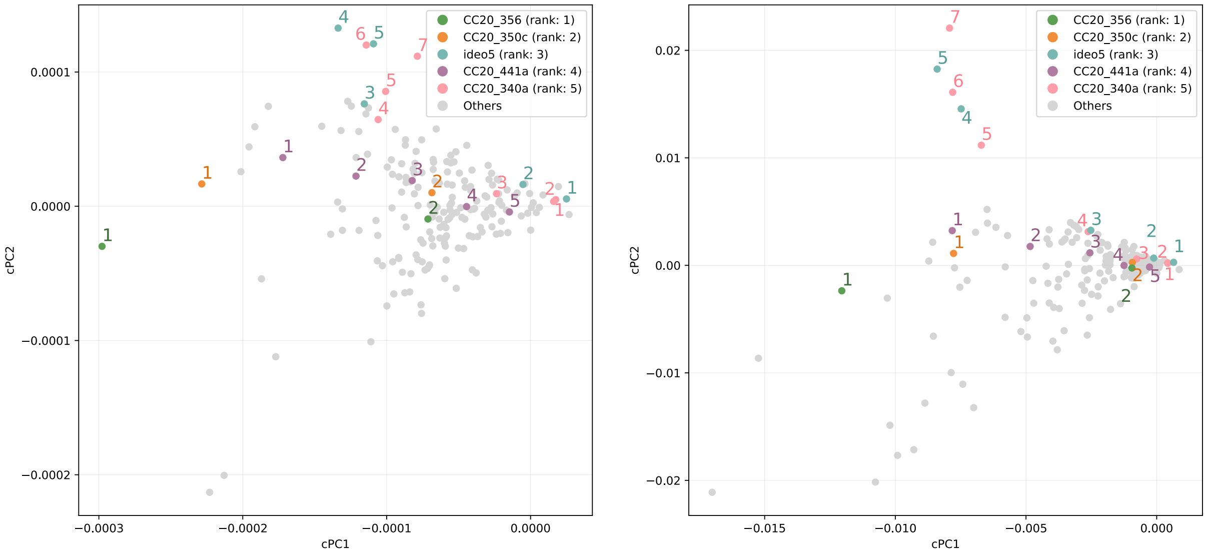

(a) Category loadings **(b)** Category coordinates

Fig. 18: Category loadings and coordinates of the top-5 variables along cPC1 (cMCA `Dem` vs `Rep`, CES 2020)

S5.4. cMCA's category loadings and coordinates (cPC2): CES 2020 (target: `Dem`, background: `Rep`)

Table 12: Variables and ranks of their value range of the categorical loadings along cPC2 (cMCA `Dem` vs `Rep`, CES 2020)

CC20.350f (rank: 1)	CC20.441a (rank: 16)	CC20.338a (rank: 31)	CC20.334g (rank: 46)	CC20.350c (rank: 61)
CC20.350g (rank: 2)	CC20.327d (rank: 17)	CC20.443.2 (rank: 32)	CC20.334e (rank: 47)	CC20.442c (rank: 62)
ideo5 (rank: 3)	CC20.355e (rank: 18)	CC20.440c (rank: 33)	CC20.355b (rank: 48)	CC20.350a (rank: 63)
CC20.340a (rank: 4)	CC20.331d (rank: 19)	CC20.332d (rank: 34)	CC20.443.5 (rank: 49)	CC20.330c (rank: 64)
CC20.320a (rank: 5)	CC20.334c (rank: 20)	CC20.331e (rank: 35)	CC20.440b (rank: 50)	CC20.334a (rank: 65)
CC20.331b (rank: 6)	CC20.350b (rank: 21)	CC20.338b (rank: 36)	CC20.442a (rank: 51)	CC20.334f (rank: 66)
CC20.442b (rank: 7)	CC20.440d (rank: 22)	CC20.334d (rank: 37)	CC20.333b (rank: 52)	CC20.350e (rank: 67)
CC20.441f (rank: 8)	CC20.441b (rank: 23)	CC20.356 (rank: 38)	CC20.332f (rank: 53)	CC20.333a (rank: 68)
CC20.441g (rank: 9)	CC20.441e (rank: 24)	CC20.442e (rank: 39)	CC20.350d (rank: 54)	CC20.330b (rank: 69)
CC20.443.4 (rank: 10)	CC20.327a (rank: 25)	CC20.302 (rank: 40)	CC20.443.3 (rank: 55)	
CC20.307 (rank: 11)	CC20.355c (rank: 26)	CC20.333d (rank: 41)	CC20.443.1 (rank: 56)	
CC20.331c (rank: 12)	CC20.332b (rank: 27)	CC20.333c (rank: 42)	CC20.355d (rank: 57)	
CC20.440a (rank: 13)	CC20.338c (rank: 28)	CC20.442d (rank: 43)	CC20.331a (rank: 58)	
CC20.332c (rank: 14)	CC20.332e (rank: 29)	CC20.332a (rank: 44)	CC20.334h (rank: 59)	
CC20.355a (rank: 15)	CC20.338d (rank: 30)	CC20.327e (rank: 45)	CC20.334b (rank: 60)	

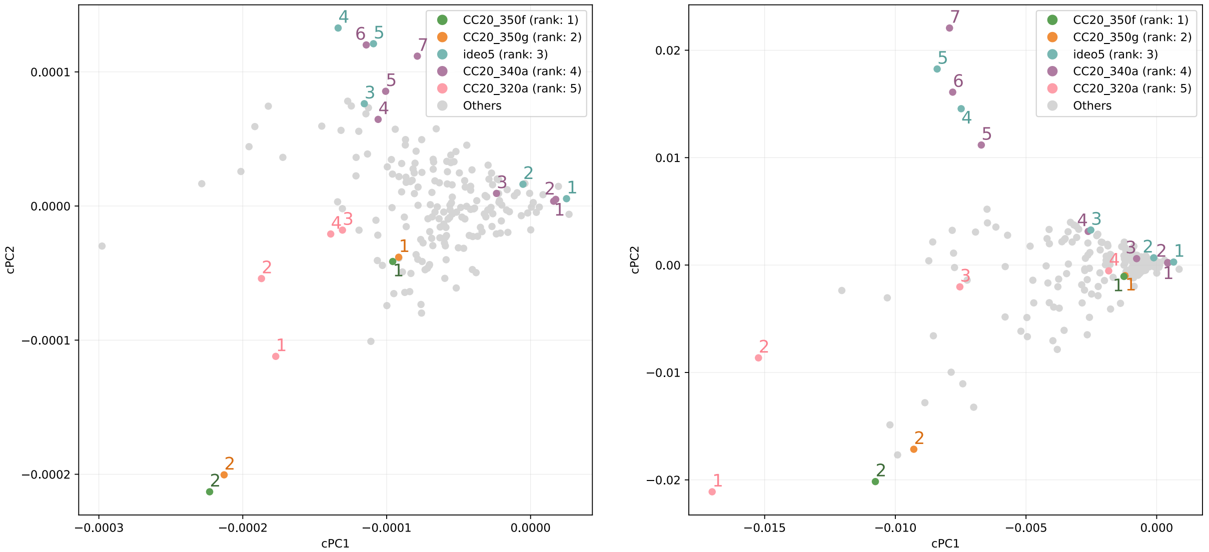

(a) Category loadings **(b)** Category coordinates

Fig. 19: Category loadings and coordinates of the top-5 variables along cPC2 (cMCA `Dem` vs `Rep`, CES 2020)

S5.5. cMCA's category loadings and coordinates of the top-5 variables (cPC1): CES 2020 (target: `Rep`, background: `Dem`)

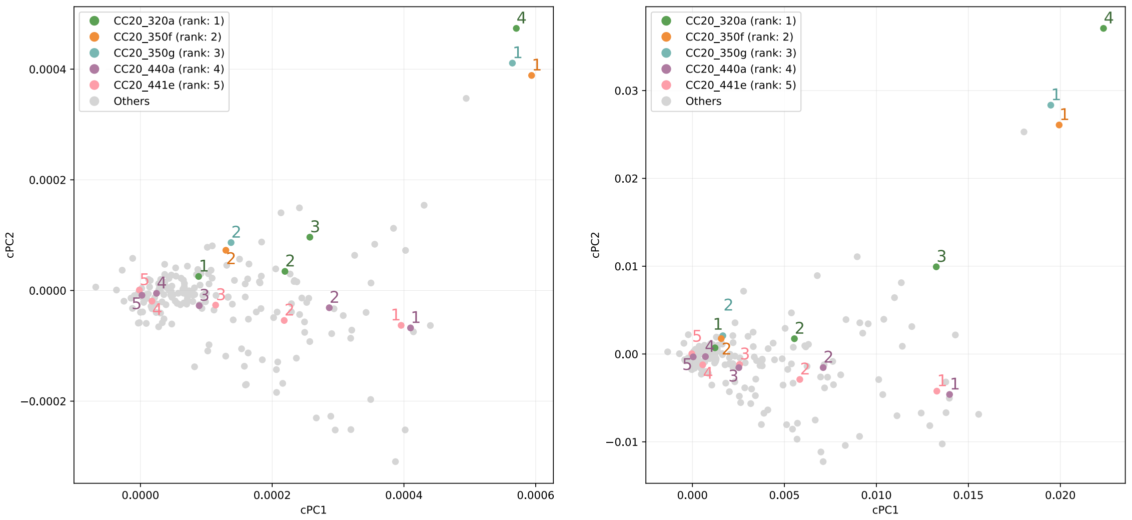

(a) Category loadings **(b)** Category coordinates

Fig. 20: Category loadings and coordinates of the top-5 variables along cPC1 (cMCA `Rep` vs `Dem`, CES 2020)

S5.6. cMCA's category loadings and coordinates (cPC2): CES 2020 (target: `Rep`, background: `Dem`)

Table 13: Variables and ranks of their value range of the categorical loadings along cPC2 (cMCA `Rep` vs `Dem`, CES 2020)

CC20.320a (rank: 1)	CC20.302 (rank: 16)	CC20.350c (rank: 31)	CC20.441g (rank: 46)	CC20.440d (rank: 61)
CC20.350g (rank: 2)	CC20.333d (rank: 17)	CC20.441e (rank: 32)	CC20.334b (rank: 47)	CC20.355b (rank: 62)
CC20.350f (rank: 3)	CC20.331e (rank: 18)	CC20.440a (rank: 33)	CC20.440b (rank: 48)	CC20.307 (rank: 63)
CC20.350b (rank: 4)	CC20.331a (rank: 19)	CC20.442c (rank: 34)	CC20.442d (rank: 49)	CC20.338b (rank: 64)
CC20.356 (rank: 5)	CC20.443.1 (rank: 20)	CC20.332d (rank: 35)	CC20.338c (rank: 50)	CC20.338a (rank: 65)
CC20.443.2 (rank: 6)	CC20.334e (rank: 21)	CC20.355a (rank: 36)	CC20.440c (rank: 51)	CC20.334d (rank: 66)
CC20.330b (rank: 7)	CC20.350e (rank: 22)	ideo5 (rank: 37)	CC20.442e (rank: 52)	CC20.332c (rank: 67)
CC20.333a (rank: 8)	CC20.334f (rank: 23)	CC20.442b (rank: 38)	CC20.334g (rank: 53)	CC20.334c (rank: 68)
CC20.327a (rank: 9)	CC20.334h (rank: 24)	CC20.332b (rank: 39)	CC20.332a (rank: 54)	CC20.332f (rank: 69)
CC20.443.3 (rank: 10)	CC20.441f (rank: 25)	CC20.327e (rank: 40)	CC20.355e (rank: 55)	
CC20.333b (rank: 11)	CC20.327d (rank: 26)	CC20.443.4 (rank: 41)	CC20.334a (rank: 56)	
CC20.350a (rank: 12)	CC20.355d (rank: 27)	CC20.355c (rank: 42)	CC20.332e (rank: 57)	
CC20.350d (rank: 13)	CC20.340a (rank: 28)	CC20.331c (rank: 43)	CC20.338d (rank: 58)	
CC20.330c (rank: 14)	CC20.441b (rank: 29)	CC20.441a (rank: 44)	CC20.331d (rank: 59)	
CC20.333c (rank: 15)	CC20.331b (rank: 30)	CC20.443.5 (rank: 45)	CC20.442a (rank: 60)	

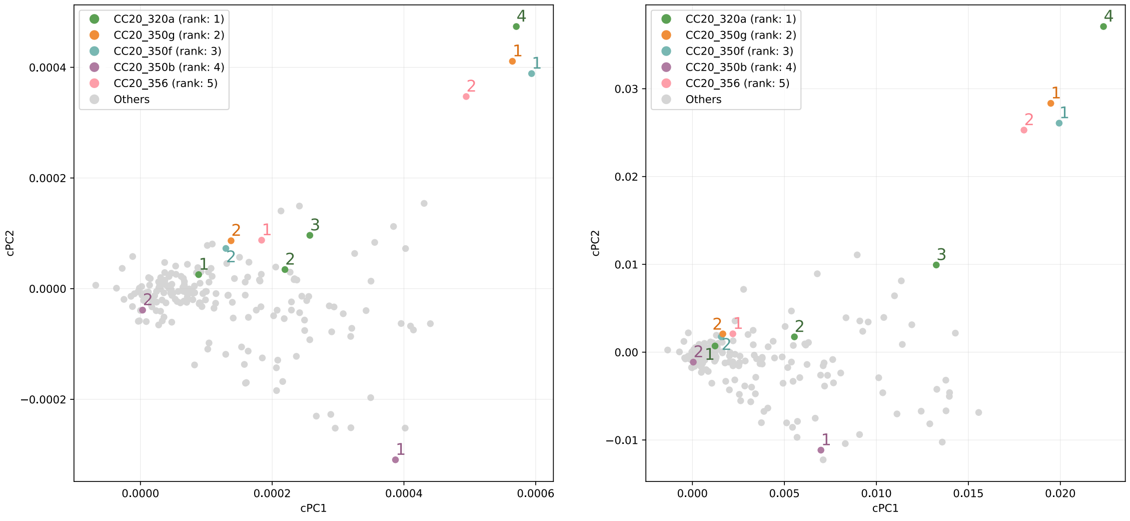

(a) Category loadings **(b)** Category coordinates

Fig. 21: Category loadings and coordinates of the top-5 variables along cPC2 (cMCA `Rep` vs `Dem`, CES 2020)

S5.7. cMCA's category loadings and coordinates of the top-5 variables (cPC1): ESS 2018 (target: `Lab`, background: `Con`)

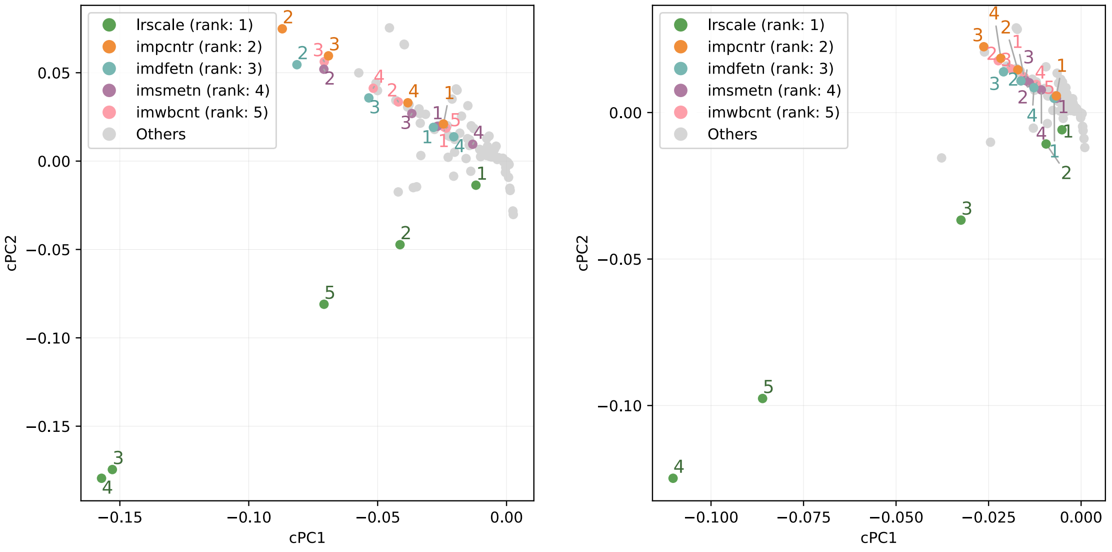

(a) Category loadings **(b)** Category coordinates

Fig. 22: Category loadings and coordinates of the top-5 variables along cPC1 (cMCA `Lab` vs `Con`, ESS 2018)

S5.8. cMCA's category loadings and coordinates (cPC2): ESS 2018 (target: `Lab`, background: `Con`)

Table 14: Variables and ranks of their value range of the categorical loadings along cPC2 (cMCA `Lab` vs `Con`, ESS 2018)

lrscale (rank: 1)	imwbcnt (rank: 6)	hmsacld (rank: 11)	ipeqopt (rank: 16)	imptrad (rank: 21)
euftf (rank: 2)	imueclt (rank: 7)	atcherp (rank: 12)	ipudrst (rank: 17)	trstun (rank: 22)
impcntr (rank: 3)	trstep (rank: 8)	freehms (rank: 13)	stfhlth (rank: 18)	impenv (rank: 23)
imsmetn (rank: 4)	imbgeco (rank: 9)	hmsfmlsh (rank: 14)	ipstrgv (rank: 19)	
imdfetn (rank: 5)	stfdem (rank: 10)	gincdif (rank: 15)	rlgdgr (rank: 20)	

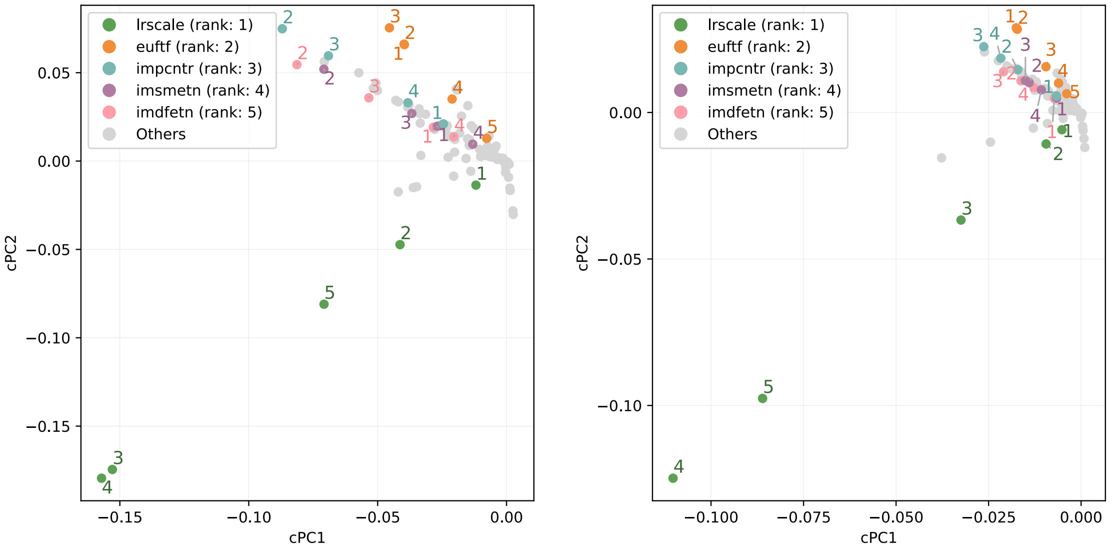

(a) Category loadings **(b)** Category coordinates

Fig. 23: Category loadings and coordinates of the top-5 variables along cPC2 (cMCA `Lab` vs `Con`, ESS 2018)

S5.9. cMCA's category loadings and coordinates of the top-5 variables (cPC1): ESS 2018 (target: `Con`, background: `Lab`)

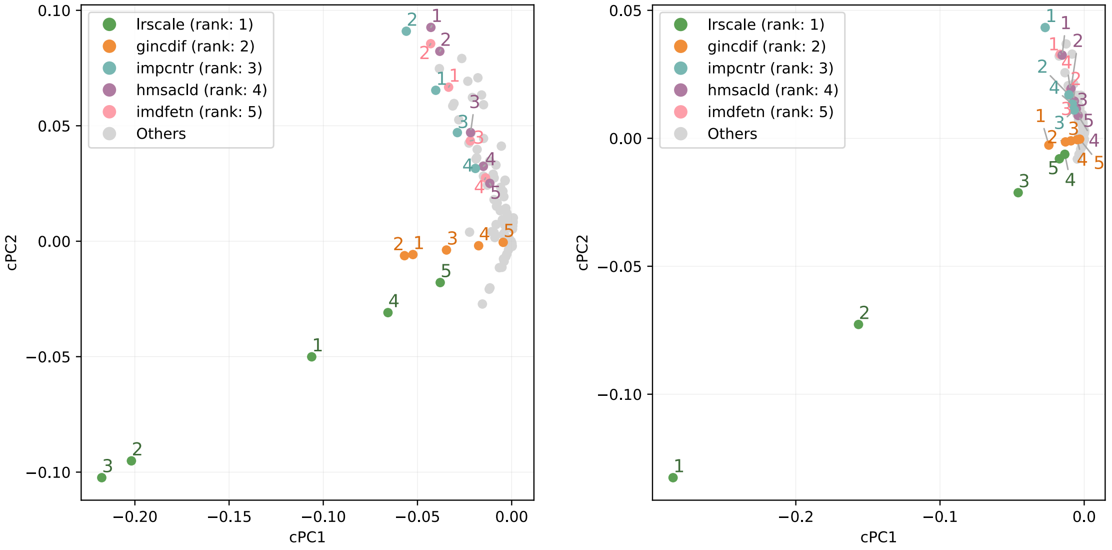

(a) Category loadings **(b)** Category coordinates

Fig. 24: Category loadings and coordinates of the top-5 variables along cPC1 (cMCA `Con` vs `Lab`, ESS 2018)

S5.10. cMCA's category loadings and coordinates (cPC2): ESS 2018 (target: `Con`, background: `Lab`)

Table 15: Variables and ranks of their value range of the categorical loadings along cPC2 (cMCA `Con` vs `Lab`, ESS 2018)

lrscale (rank: 1)	imwbcnt (rank: 6)	euftf (rank: 11)	ipudrst (rank: 16)	trstun (rank: 21)
hmsacld (rank: 2)	imueclt (rank: 7)	imbgeco (rank: 12)	gincdif (rank: 17)	imptrad (rank: 22)
impcntr (rank: 3)	freehms (rank: 8)	stfdem (rank: 13)	rlgdgr (rank: 18)	impenv (rank: 23)
imdfetn (rank: 4)	hmsfmlsh (rank: 9)	atcherp (rank: 14)	ipeqopt (rank: 19)	
imsmetn (rank: 5)	trstep (rank: 10)	stfhlth (rank: 15)	ipstrgv (rank: 20)	

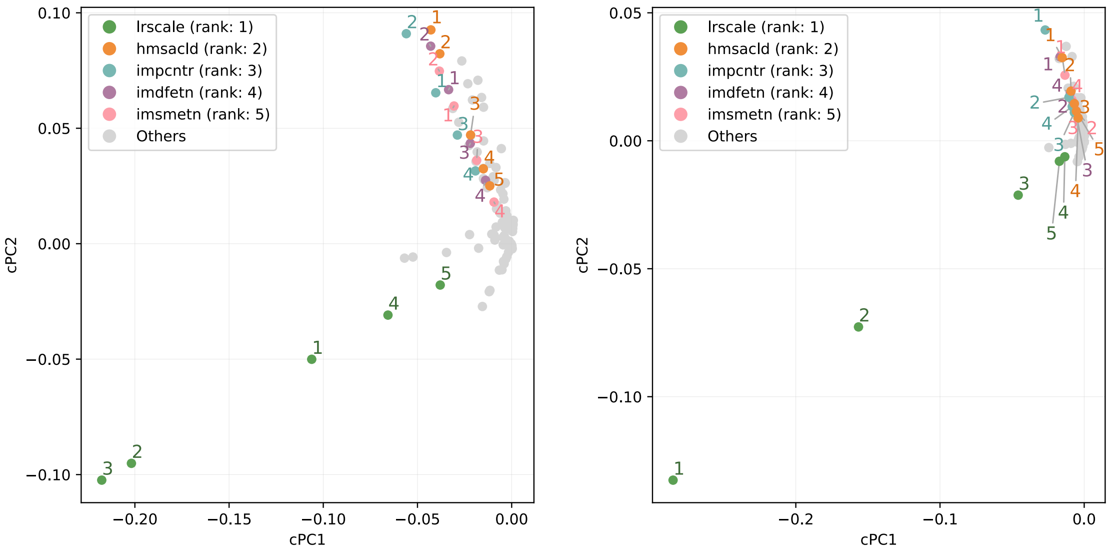

(a) Category loadings

(b) Category coordinates

Fig. 25: Category loadings and coordinates of the top-5 variables along cPC2 (cMCA `Con` vs `Lab`, ESS 2018)

S5.11. cMCA's category loadings and coordinates of the top-5 variables (cPC1): ESS 2018 (target: `Lab`, background: `UKIP`)

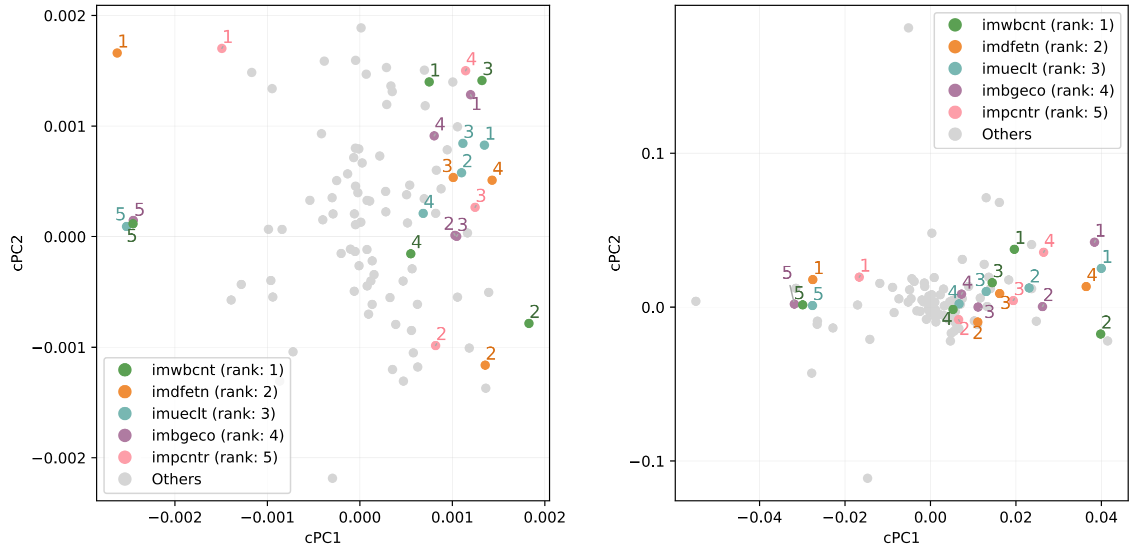

(a) Category loadings　　**(b)** Category coordinates

Fig. 26: Category loadings and coordinates of the top-5 variables along cPC1 (cMCA `Lab` vs `UKIP`, ESS 2018)

S5.12. cMCA's category loadings and coordinates (cPC2): ESS 2018 (target: `Lab`, background: `UKIP`)

Table 16: Variables and ranks of their value range of the categorical loadings along cPC2 (cMCA `Lab` vs `UKIP`, ESS 2018)

lrscale (rank: 1)	impcntr (rank: 6)	imwbcnt (rank: 11)	atcherp (rank: 16)	imueclt (rank: 21)
hmsacld (rank: 2)	ipstrgv (rank: 7)	freehms (rank: 12)	imbgeco (rank: 17)	ipeqopt (rank: 22)
imsmetn (rank: 3)	trstep (rank: 8)	stfdem (rank: 13)	imptrad (rank: 18)	ipudrst (rank: 23)
hmsfmlsh (rank: 4)	rlgdgr (rank: 9)	euftf (rank: 14)	impenv (rank: 19)	
imdfetn (rank: 5)	gincdif (rank: 10)	trstun (rank: 15)	stfhlth (rank: 20)	

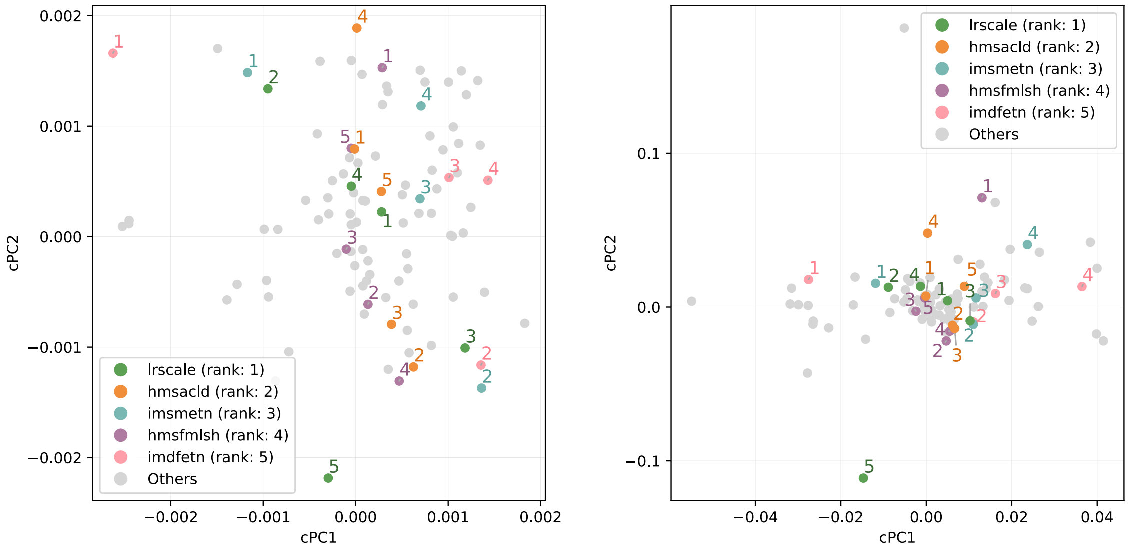

(a) Category loadings **(b)** Category coordinates

Fig. 27: Category loadings and coordinates of the top-5 variables along cPC2 (cMCA `Lab` vs `UKIP`, ESS 2018)

S5.13. cMCA's category loadings and coordinates of the top-5 variables (cPC1): ESS 2018 (target: `Con`, background: `UKIP`)

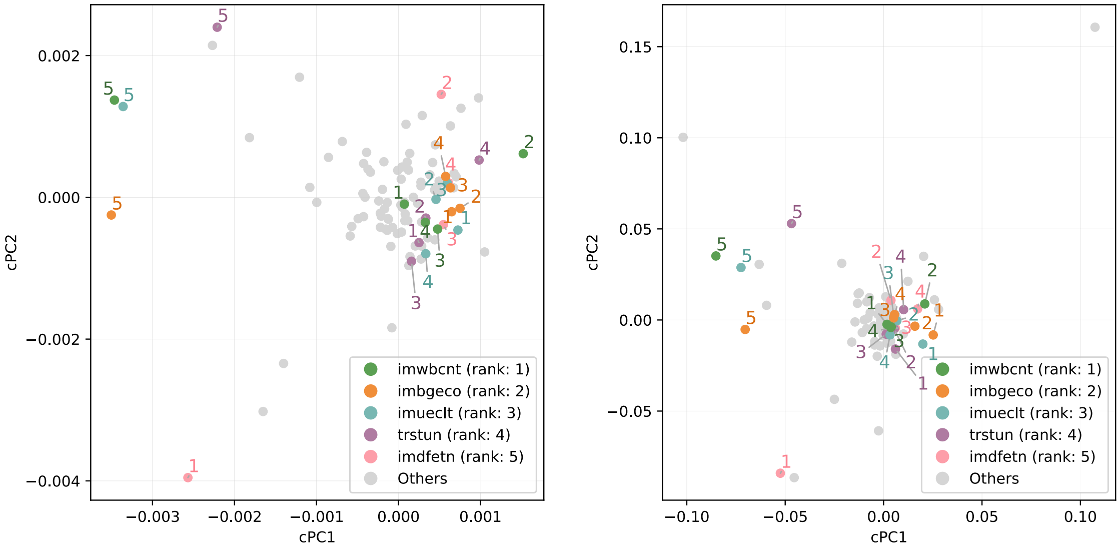

(a) Category loadings **(b)** Category coordinates

Fig. 28: Category loadings and coordinates of the top-5 variables along cPC1 (cMCA `Con` vs `UKIP`, ESS 2018)

S5.14. cMCA's category loadings and coordinates (cPC2): ESS 2018 (target: `Con`, background: `UKIP`)

Table 17: Variables and ranks of their value range of the categorical loadings along cPC2 (cMCA `Con` vs `UKIP`, ESS 2018)

imdfetn (rank: 1)	trstep (rank: 6)	imwbcnt (rank: 11)	hmsacld (rank: 16)	imbgeco (rank: 21)
impcntr (rank: 2)	stfdem (rank: 7)	ipstrgv (rank: 12)	euftf (rank: 17)	ipudrst (rank: 22)
imsmetn (rank: 3)	imueclt (rank: 8)	hmsfmlsh (rank: 13)	imptrad (rank: 18)	impenv (rank: 23)
trstun (rank: 4)	gincdif (rank: 9)	stfhlth (rank: 14)	rlgdgr (rank: 19)	
lrscale (rank: 5)	atcherp (rank: 10)	freehms (rank: 15)	ipeqopt (rank: 20)	

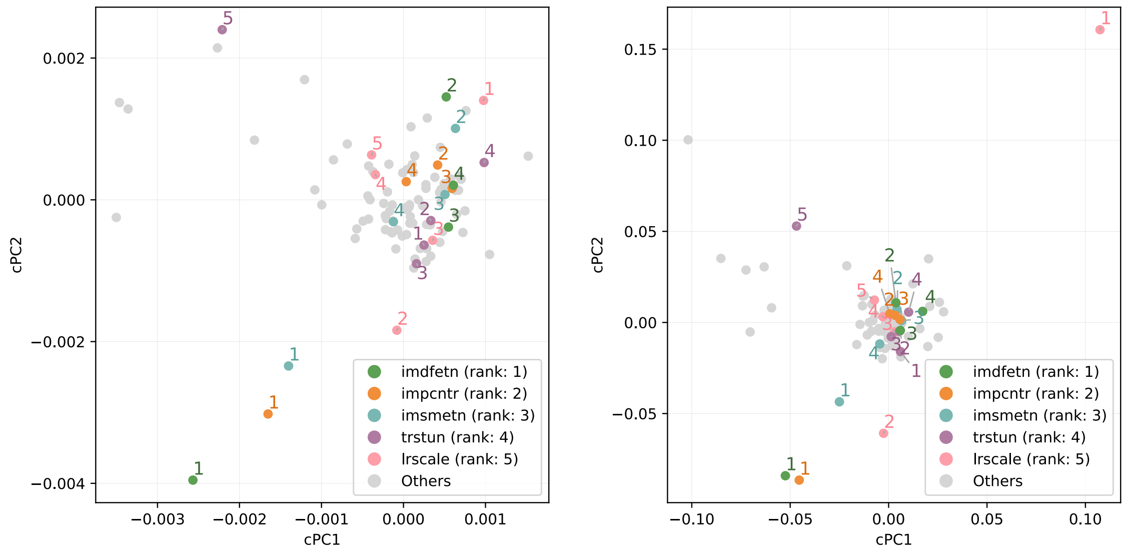

(a) Category loadings **(b)** Category coordinates

Fig. 29: Category loadings and coordinates of the top-5 variables along cPC2 (cMCA `Con` vs `UKIP`, ESS 2018)
