# Supplementary material for: Contrastive multiple correspondence analysis (cMCA): Using contrastive learning to identify latent subgroups in political parties
Source: PLoS One. 2023 Jul 10;18(7):e0287180. doi: 10.1371/journal.pone.0287180 (PMC10332614; doi:10.1371/journal.pone.0287180)
Supplement: S6 Appendix — (PDF) [file pone.0287180.s006.pdf]

# S6. Variable Coding Scheme

## *S6.1. Variable Coding Scheme for the CES 2020 Survey*

***Seven-Point Ideology*** (`CC20.340a`): This is an ordinal variable measuring respondents' self-identified ideologies on the liberal-conservative scale. This variable is originally coded from a scale of 1 (very liberal) to 7 (very conservative).

***Five-Point Ideology*** (`ideo5`): This is an ordinal variable measuring respondents' self-identified ideologies on the liberal-conservative scale. This variable is originally coded from a scale of 1 (very liberal) to 5 (very conservative).

***PartyID*** (`CC20.433a`): This is a categorical variable measuring respondents' self-identified partisanship. This variable is originally coded by parties: 1 refers to Democrat, 2 refers to Republican, 3 refers to independent, and 4 refers to others.

***Feeling about Police*** (`CC20.307`): This is an ordinal variable measuring respondents' self-identified feelings about the police. This variable is originally coded from a scale of 1 (mostly safe) to 4 (mostly unsafe).

***Feeling about the Nation's Economy Last Year*** (`CC20.302`): This is an ordinal variable measuring respondents' self-identified feelings about the nation's economy over the past year. This variable is originally coded from a scale of 1 (gotten much better) to 5 (gotten much worse).

***Trump's Job Approval*** (`CC20.320a`): This is an ordinal variable measuring respondents' self-identified feelings about Trump's performance. This variable is originally coded from a scale of 1 (strongly approve) to 4 (strongly disapprove).

***Expanding Medicare to Everybody*** (`CC20.327a`): This is a binary variable measuring respondents' self-identified support about expanding medicare to a single comprehensive public health care coverage program that would cover all Americans. This variable is originally coded as: 1 (support) and 2 (oppose).

***Health Care for All*** (`CC20.327d`): This is a binary variable measuring respondents' self-

identified support about repealing the entire Affordable Care Act. This variable is originally coded as: 1 (support) and 2 (oppose).

***Restoring Obamacare*** (`CC20.327e`): This is a binary variable measuring respondents' self-identified support about restoring the Affordable Care Act's mandate that all individuals be required to purchase health insurance. This variable is originally coded as: 1 (support) and 2 (oppose).

***Banning Assault Rifles*** (`CC20.330b`): This is a binary variable measuring respondents' self-identified support about banning assault rifles. This variable is originally coded as: 1 (support) and 2 (oppose).

***Making Concealed-Carry Permit Easier*** (`CC20.330c`): This is a binary variable measuring respondents' self-identified support about making concealed-carry permit easier. This variable is originally coded as: 1 (support) and 2 (oppose).

***Granting Legal Status to all "Behaved" Illegal Immigrants*** (`CC20.331a`): This is a binary variable measuring respondents' self-identified support about granting illegal immigrants who hold jobs, pay taxes at least three years, and have not been committed to any felony crimes. This variable is originally coded as: 1 (support) and 2 (oppose).

***Increasing the Number of Border Patrols*** (`CC20.331b`): This is a binary variable measuring respondents' self-identified support about increasing the number of border patrols on the US-Mexican border. This variable is originally coded as: 1 (support) and 2 (oppose).

***Withholding Federal Funds from the Police Departments that Fail to Report Identified Illegal Immigrants*** (`CC20.331c`): This is a binary variable measuring respondents' self-identified support about withholding federal funds from any local police department that does not report to the federal government anyone they identify as an illegal immigrant. This variable is originally coded as: 1 (support) and 2 (oppose).

***Eliminating the Visa Lottery and Ending Family-Based Migration*** (`CC20.331d`): This is a binary variable measuring respondents' self-identified support about decreasing legal immigration by fifty percent over the next ten years by eliminating the visa lottery

and ending family-based migration. This variable is originally coded as: 1 (support) and 2 (oppose).

***Increasing Spending on Border Security*** (`CC20.331e`): This is a binary variable measuring respondents' self-identified support about increasing spending on border security by $25 billion, including building a wall between the U.S. and Mexico. This variable is originally coded as: 1 (support) and 2 (oppose).

***Partial Permission of Abortion*** (`CC20.332a`): This is a binary variable measuring respondents' self-identified support about permitting abortion only in case of rape, incest or when the woman's life is in danger. This variable is originally coded as: 1 (support) and 2 (oppose).

***The Twentieth Week Prohibition*** (`CC20.332b`): This is a binary variable measuring respondents' self-identified support about prohibiting all abortions after the twentieth week of pregnancy. This variable is originally coded as: 1 (support) and 2 (oppose).

***Insurance Coverage*** (`CC20.332c`): This is a binary variable measuring respondents' self-identified support about allowing employers to decline coverage of abortions in insurance plans. This variable is originally coded as: 1 (support) and 2 (oppose).

***Prohibiting Funds*** (`CC20.332d`): This is a binary variable measuring respondents' self-identified support about prohibiting the expenditure of funds authorized or appropriated by federal law for any abortion. This variable is originally coded as: 1 (support) and 2 (oppose).

***Illegalizing Abortion*** (`CC20.332e`): This is a binary variable measuring respondents' self-identified support about making abortions illegal in all circumstances. This variable is originally coded as: 1 (support) and 2 (oppose).

***Where to Abortion*** (`CC20.332f`): This is a binary variable measuring respondents' self-identified support about prohibiting states from requiring that abortions be performed only at hospitals (not clinics). This variable is originally coded as: 1 (support) and 2 (oppose).

***Empowering Environmental Protection Agency*** (`CC20.333a`): This is a binary variable measuring respondents' self-identified support about giving the Environmental Protec-

tion Agency power to regulate Carbon Dioxide emissions. This variable is originally coded as: 1 (support) and 2 (oppose).

***Minimum Amount of Renewable Fuels*** (`CC20.333b`): This is a binary variable measuring respondents' self-identified support about requiring that each state use a minimum amount of renewable fuels for electricity generation even if electricity prices increase a little. This variable is originally coded as: 1 (support) and 2 (oppose).

***Minimum Amount of Renewable Fuels*** (`CC20.333b`): This is a binary variable measuring respondents' self-identified support about requiring that each state use a minimum amount of renewable fuels for electricity generation even if electricity prices increase a little. This variable is originally coded as: 1 (support) and 2 (oppose).

***Strengthening Enforcement of the Clean Air Act and Clean Water Ac*** (`CC20.333c`): This is a binary variable measuring respondents' self-identified support about strengthening the Environmental Protection Agency enforcement of the Clean Air Act and Clean Water Act even if it costs U.S. jobs. This variable is originally coded as: 1 (support) and 2 (oppose).

***Raising Fuel Efficiency*** (`CC20.333d`): This is a binary variable measuring respondents' self-identified support about raising the average fuel efficiency for all cars and trucks by 2025. This variable is originally coded as: 1 (support) and 2 (oppose).

***Nonviolent Drug Offenders*** (`CC20.334a`): This is a binary variable measuring respondents' self-identified support about eliminating mandatory minimum sentences for non- violent drug offenders. This variable is originally coded as: 1 (support) and 2 (oppose).

***Body Camera Policy*** (`CC20.334b`): This is a binary variable measuring respondents' self-identified support about requiring police officers to wear body cameras that record all of their activities while on duty. This variable is originally coded as: 1 (support) and 2 (oppose).

***Increasing the Number of Police*** (`CC20.334c`): This is a binary variable measuring respondents' self-identified support about increasing the number of police on the street by ten percent, even if it means fewer funds for other public services. This variable is originally

coded as: 1 (support) and 2 (oppose).

***Decreasing the Number of Police*** (`CC20.334d`): This is a binary variable measuring respondents' self-identified support about decreasing the number of police on the street by ten percent, and increase funding for other public services. This variable is originally coded as: 1 (support) and 2 (oppose).

***Banning Choke Holds*** (`CC20.334e`): This is a binary variable measuring respondents' self-identified support about banning the use of choke holds by police. This variable is originally coded as: 1 (support) and 2 (oppose).

***Registry of Investigated Police*** (`CC20.334f`): This is a binary variable measuring respondents' self-identified support about creating a national registry of police who have been investigated for or disciplined for misconduct. This variable is originally coded as: 1 (support) and 2 (oppose).

***Sending Surplus Weapons to Police Departments*** (`CC20.334g`): This is a binary variable measuring respondents' self-identified support about ending the Department of Defense program that sends surplus military weapons and equipment to police departments. This variable is originally coded as: 1 (support) and 2 (oppose).

***Suing Recklessly Disregarded*** (`CC20.334h`): This is a binary variable measuring respondents' self-identified support about allowing individuals or their families to sue a police officer for damages if the officer is found to have "recklessly disregarded" the individual's rights. This variable is originally coded as: 1 (support) and 2 (oppose).

***Tariffs on China*** (`CC20.338a`): This is a binary variable measuring respondents' self-identified support about tariffs on $200 billion worth of goods imported from China. This variable is originally coded as: 1 (support) and 2 (oppose).

***Tariff Barriers I*** (`CC20.338b`): This is a binary variable measuring respondents' self-identified support about 25% tariffs on imported steel and 10% on imported aluminum, EXCEPT from Canada and Mexico. This variable is originally coded as: 1 (support) and 2 (oppose).

***Tariff Barriers II*** (`CC20.338c`): This is a binary variable measuring respondents' self-identified support about 25% tariffs on all imported steel and 10% on imported aluminum, INCLUDING from Canada and Mexico. This variable is originally coded as: 1 (support) and 2 (oppose).

***Tariffs on the Europe*** (`CC20.338d`): This is a binary variable measuring respondents' self-identified support about increasing tariffs on European aircraft and agricultural products. This variable is originally coded as: 1 (support) and 2 (oppose).

***Prohibiting Discrimination on Gender Identity*** (`CC20.350a`): This is a binary variable measuring respondents' self-identified support about amending federal laws to prohibit discrimination on the basis of gender identity and sexual orientation. This variable is originally coded as: 1 (favor) and 2 (oppose).

***Raising the Minimum Wage*** (`CC20.350b`): This is a binary variable measuring respondents' self-identified support about raising the minimum wage to $15 an hour. This variable is originally coded as: 1 (favor) and 2 (oppose).

***Confirming Brett Kavanaugh*** (`CC20.350c`): This is a binary variable measuring respondents' self-identified support about confirming Brett Kavanaugh to become a Justice of the Supreme Court of the United States. This variable is originally coded as: 1 (favor) and 2 (oppose).

***Equal Pay*** (`CC20.350d`): This is a binary variable measuring respondents' self-identified support about requiring equal pay for women and men who are doing similar jobs and have similar qualifications. This variable is originally coded as: 1 (favor) and 2 (oppose).

***The Dream Act*** (`CC20.350e`): This is a binary variable measuring respondents' self-identified support about providing permanent resident status to children of immigrants who were brought to the United States, and a pathway to citizenship if the citizenship requirements are met. This variable is originally coded as: 1 (favor) and 2 (oppose).

***Removing President Trump I*** (`CC20.350f`): This is a binary variable measuring respondents' self-identified support about removing President Trump from office for abuse of

power. This variable is originally coded as: 1 (favor) and 2 (oppose).

***Removing President Trump II*** (`CC20.350g`): This is a binary variable measuring respondents' self-identified support about removing President Trump from office for obstruction of Congress. This variable is originally coded as: 1 (favor) and 2 (oppose).

***Paris Climate Agreement*** (`CC20.355a`): This is a binary variable measuring respondents' self-identified support about withdrawing the United States from the Paris Climate Agreement. This variable is originally coded as: 1 (support) and 2 (oppose).

***Trans-Pacific Partnership Trade Agreement*** (`CC20.355b`): This is a binary variable measuring respondents' self-identified support about withdrawing the United States from the Trans-Pacific Partnership trade agreement. This variable is originally coded as: 1 (support) and 2 (oppose).

***Clean Power Plant Rules*** (`CC20.355c`): This is a binary variable measuring respondents' self-identified support about repealing the Clean Power Plant Rules. This variable is originally coded as: 1 (support) and 2 (oppose).

***Transgender in the Military*** (`CC20.355d`): This is a binary variable measuring respondents' self-identified support about banning transgender people in the military. This variable is originally coded as: 1 (support) and 2 (oppose).

***Food Stamp*** (`CC20.355e`): This is a binary variable measuring respondents' self-identified support about requiring able-bodied adults 18 to 49 years of age who do not have dependents to have a job in order to receive food stamps. This variable is originally coded as: 1 (support) and 2 (oppose).

***Amy Coney Barrett*** (`CC20.356`): This is a binary variable measuring respondents' self-identified support about confirming Amy Coney Barrett. This variable is originally coded as: 1 (support) and 2 (oppose).

***White Privilege*** (`CC20.440a`): This is an ordinary variable measuring respondents' opinion about white people in the U.S. have certain advantages because of the color of their skin. This variable is originally coded from a scale of 1 (strongly agree) to 5 (strongly disagree).

***Racial Problems*** (`CC20.440b`): This is an ordinary variable measuring respondents' opinion about racial problems in the U.S. are rare, isolated situations. This variable is originally coded from a scale of 1 (strongly agree) to 5 (strongly disagree).

***Women Empowerment*** (`CC20.440c`): This is an ordinary variable measuring respondents' opinion about women seek to gain power by getting control over men. This variable is originally coded from a scale of 1 (strongly agree) to 5 (strongly disagree).

***Women Stereotype*** (`CC20.440d`): This is an ordinary variable measuring respondents' opinion about women are too easily offended. This variable is originally coded from a scale of 1 (strongly agree) to 5 (strongly disagree).

***Blacks Are Like Others*** (`CC20.440a`): This is an ordinary variable measuring respondents' opinion about blacks should do the same as other minorities (e.g., Irish, Italians, and Jewish ) without any special favors to overcome prejudice. This variable is originally coded from a scale of 1 (strongly agree) to 5 (strongly disagree).

***Impacts of Slavery*** (`CC20.440b`): This is an ordinary variable measuring respondents' opinion about generations of slavery and discrimination have created conditions that make it difficult for blacks to work their way out of the lower class. This variable is originally coded from a scale of 1 (strongly agree) to 5 (strongly disagree).

***Existence of Racial Discrimination*** (`CC20.440e`): This is an ordinary variable measuring respondents' opinion about being resented when Whites deny the existence of racial discrimination. This variable is originally coded from a scale of 1 (strongly agree) to 5 (strongly disagree).

***Racial Differences I*** (`CC20.440f`): This is an ordinary variable measuring respondents' opinion about whites do not go to great lengths to understand the problems African Americans face. This variable is originally coded from a scale of 1 (strongly agree) to 5 (strongly disagree).

***Racial Differences II*** (`CC20.440g`): This is an ordinary variable measuring respondents' opinion about whites get away with offenses that African Americans would never get away

with. This variable is originally coded from a scale of 1 (strongly agree) to 5 (strongly disagree).

***Assassination of Qasem Soleimani*** (`CC20.442a`): This is a binary variable measuring respondents' self-identified support about assassination of Qasem Soleimani. This variable is originally coded as: 1 (support) and 2 (oppose).

***Iranian Sanction*** (`CC20.442b`): This is a binary variable measuring respondents' self-identified support about withdrawing the United States from the Iran Nuclear Accord and reimpose sanctions on Iran. This variable is originally coded as: 1 (support) and 2 (oppose).

***Border Wall*** (`CC20.442c`): This is a binary variable measuring respondents' self-identified support about declaring a national emergency to permit construction of border wall with Mexico. This variable is originally coded as: 1 (support) and 2 (oppose).

***The Asylum Program*** (`CC20.442d`): This is a binary variable measuring respondents' self-identified support about suspending a program that allows migrants to remain in the US while their asylum cases were being decided. This variable is originally coded as: 1 (support) and 2 (oppose).

***Kurdish Support*** (`CC20.442e`): This is a binary variable measuring respondents' self-identified support about withdrawing troops from Kurdish-controlled region of northern Syria on the border with Turkey. This variable is originally coded as: 1 (support) and 2 (oppose).

***State Legislature Spending I*** (`CC20.443.1`): This is an ordinary variable measuring respondents' opinion about the state legislature to spend money on welfare. This variable is originally coded from a scale of 1 (greatly increase) to 5 (greatly decrease).

***State Legislature Spending II*** (`CC20.443.2`): This is an ordinary variable measuring respondents' opinion about the state legislature to spend money on health care. This variable is originally coded from a scale of 1 (greatly increase) to 5 (greatly decrease).

***State Legislature Spending III*** (`CC20.443.3`): This is an ordinary variable measuring respondents' opinion about the state legislature to spend money on education. This variable

is originally coded from a scale of 1 (greatly increase) to 5 (greatly decrease).

***State Legislature Spending IV*** (`CC20.443.4`): This is an ordinary variable measuring respondents' opinion about the state legislature to spend money on law enforcement. This variable is originally coded from a scale of 1 (greatly increase) to 5 (greatly decrease).

***State Legislature Spending V*** (`CC20.443.5`): This is an ordinary variable measuring respondents' opinion about the state legislature to spend money on transportation/infrastructure. This variable is originally coded from a scale of 1 (greatly increase) to 5 (greatly decrease).

### *S6.2. Variable Coding Scheme for the ESS 2018 Survey*

***Left-Right Scale*** (`lrscale`): This is an ordinal variable measuring respondents' self-identified ideologies on the left-right scale. This variable is originally coded from a scale of 0 (left) to 10 (right). We further recode this variable as a five-point scale: respondents who originally responded as 0 or 1 are recoded as 1, those who originally responded as 2 or 3 are recoded as 2, those who originally responded as 4, 5, or 6 are recoded as 3, those who originally responded as 7 or 8 are recoded as 4, and those who originally responded as 9 or 10 are recoded as 5.

***Trust in the European Parliament*** (`trstep`): This is an ordinal variable measuring respondents' self-identified trust in the European Parliament. This variable is originally coded from a scale of 0 (not trust at all) to 10 (completely trust). We further recode this variable as a five-point scale: respondents who originally responded as 0 or 1 are recoded as 1, those who originally responded as 2 or 3 are recoded as 2, those who originally responded as 4, 5, or 6 are recoded as 3, those who originally responded as 7 or 8 are recoded as 4, and those who originally responded as 9 or 10 are recoded as 5.

***Trust in the United Nations*** (`trstun`): This is an ordinal variable measuring respondents' self-identified trust in the United Nations. This variable is originally coded from a scale of 0 (not trust at all) to 10 (completely trust). We further recode this variable as a five-point scale: respondents who originally responded as 0 or 1 are recoded as 1, those who originally responded as 2 or 3 are recoded as 2, those who originally responded as 4, 5, or 6 are recoded as 3, those who originally responded as 7 or 8 are recoded as 4, and those who originally responded as 9 or 10 are recoded as 5.

***Democracy Satisfaction*** (`stfdem`): This is an ordinal variable measuring respondents' self-identified satisfaction with how democracy works in the U.K. This variable is originally coded from a scale of 0 (extremely dissatisfied) to 10 (extremely satisfied). We further recode this variable as a five-point scale: respondents who originally responded as 0 or 1 are recoded as 1, those who originally responded as 2 or 3 are recoded as 2, those who originally

responded as 4, 5, or 6 are recoded as 3, those who originally responded as 7 or 8 are recoded as 4, and those who originally responded as 9 or 10 are recoded as 5.

***State of Health in the U.K.*** (`stfhlth`): This is an ordinal variable measuring respondents' self-identified feeling about the state of health services in the U.K. nowadays. This variable is originally coded from a scale of 0 (extremely dissatisfied) to 10 (extremely satisfied). We further recode this variable as a five-point scale: respondents who originally responded as 0 or 1 are recoded as 1, those who originally responded as 2 or 3 are recoded as 2, those who originally responded as 4, 5, or 6 are recoded as 3, those who originally responded as 7 or 8 are recoded as 4, and those who originally responded as 9 or 10 are recoded as 5.

***Income Inequality*** (`gincdif`): This is an ordinal variable measuring respondents' self-identified agreement with that governments should reduce differences in income levels. This variable is originally coded from a scale of 1 (agree strongly) to 5 (disagree strongly).

***Gay Life*** (`freehms`): This is an ordinal variable measuring respondents' self-identified agreement with that gays and lesbians free to live life as they wish. This variable is originally coded from a scale of 1 (agree strongly) to 5 (disagree strongly).

***Gay Shame*** (`hmsfmlsh`): This is an ordinal variable measuring respondents' self-identified agreement with that respondents are shamed if close family member is gay or lesbian. This variable is originally coded from a scale of 1 (agree strongly) to 5 (disagree strongly).

***Gay Adoption*** (`hmsacld`): This is an ordinal variable measuring respondents' self-identified agreement with that gay and lesbian couples adopt children. This variable is originally coded from a scale of 1 (agree strongly) to 5 (disagree strongly).

***European Unification*** (`euftf`): This is an ordinal variable measuring respondents' self-identified feelings about European Union Unification between it has gone too far and it should go further. This variable is originally coded from a scale of 0 (gone too far) to 10 (going further). We further recode this variable as a five-point scale: respondents who originally responded as 0 or 1 are recoded as 1, those who originally responded as 2 or 3

are recoded as 2, those who originally responded as 4, 5, or 6 are recoded as 3, those who originally responded as 7 or 8 are recoded as 4, and those who originally responded as 9 or 10 are recoded as 5.

***Same Race Immigrants*** (`imsmetn`): This is an ordinal variable measuring respondents' self-identified feelings about allowing immigrants of the same race as the majority. This variable is originally coded from a scale of 1 (allow many) to 5 (allow none).

***Different Race Immigrants*** (`imdfetn`): This is an ordinal variable measuring respondents' self-identified feelings about allowing immigrants of the different race as non-majority. This variable is originally coded from a scale of 1 (allow many) to 5 (allow none).

***Non-European & Poor Immigrants*** (`impcntr`): This is an ordinal variable measuring respondents' self-identified feelings about allowing immigrants who are poor and from non-European countries. This variable is originally coded from a scale of 1 (allow many) to 5 (allow none).

***Immigration to Economy*** (`imbgeco`): This is an ordinal variable measuring respondents' opinions about immigration's impacts on local economy. This variable is originally coded from a scale of 0 (bad for economy) to 10 (good for economy). We further recode this variable as a five-point scale: respondents who originally responded as 0 or 1 are recoded as 1, those who originally responded as 2 or 3 are recoded as 2, those who originally responded as 4, 5, or 6 are recoded as 3, those who originally responded as 7 or 8 are recoded as 4, and those who originally responded as 9 or 10 are recoded as 5.

***Immigration to Culture*** (`imueclt`): This is an ordinal variable measuring respondents' opinions about whether immigration undermines or enriches local cultural life. This variable is originally coded from a scale of 0 (undermining) to 10 (enriching). We further recode this variable as a five-point scale: respondents who originally responded as 0 or 1 are recoded as 1, those who originally responded as 2 or 3 are recoded as 2, those who originally responded as 4, 5, or 6 are recoded as 3, those who originally responded as 7 or 8 are recoded as 4, and those who originally responded as 9 or 10 are recoded as 5.

***Immigration to Living Quality*** (`imwbcnt`): This is an ordinal variable measuring respondents' opinions about whether immigration makes the U.K. a worse or better place to live. This variable is originally coded from a scale of 0 (worse) to 10 (better). We further recode this variable as a five-point scale: respondents who originally responded as 0 or 1 are recoded as 1, those who originally responded as 2 or 3 are recoded as 2, those who originally responded as 4, 5, or 6 are recoded as 3, those who originally responded as 7 or 8 are recoded as 4, and those who originally responded as 9 or 10 are recoded as 5.

***Emotionally Attached to Europe*** (`atcherp`): This is an ordinal variable measuring respondents' feelings about how emotionally they are attached to Europe. This variable is originally coded from a scale of 0 (not at all) to 10 (very). We further recode this variable as a five-point scale: respondents who originally responded as 0 or 1 are recoded as 1, those who originally responded as 2 or 3 are recoded as 2, those who originally responded as 4, 5, or 6 are recoded as 3, those who originally responded as 7 or 8 are recoded as 4, and those who originally responded as 9 or 10 are recoded as 5.

***Religiosity*** (`rlgdgr`): This is an ordinal variable measuring respondents' religious levels. This variable is originally coded from a scale of 0 (not at all) to 10 (very). We further recode this variable as a five-point scale: respondents who originally responded as 0 or 1 are recoded as 1, those who originally responded as 2 or 3 are recoded as 2, those who originally responded as 4, 5, or 6 are recoded as 3, those who originally responded as 7 or 8 are recoded as 4, and those who originally responded as 9 or 10 are recoded as 5.

***Equality*** (`ipeqopt`): This is an ordinal variable measuring respondents' opinions about that it is important that people are treated equally and have equal opportunities. This variable is originally coded from a scale of 1 (very much like me) to 6 (not like me at all). We further recode this variable as a three-point scale: respondents who originally responded as 1 or 2 are recoded as 1, those who originally responded as 3 or 4 are recoded as 2, and those who originally responded as 5 or 6 are recoded as 3.

***Understanding*** (`ipudrst`): This is an ordinal variable measuring respondents' opinions

about that it is important to understand different people. This variable is originally coded from a scale of 1 (very much like me) to 6 (not like me at all). We further recode this variable as a three-point scale: respondents who originally responded as 1 or 2 are recoded as 1, those who originally responded as 3 or 4 are recoded as 2, and those who originally responded as 5 or 6 are recoded as 3.

***Strong Government*** (`ipstrgv`): This is an ordinal variable measuring respondents' opinions about that it is important that government is strong and ensures safety. This variable is originally coded from a scale of 1 (very much like me) to 6 (not like me at all). We further recode this variable as a three-point scale: respondents who originally responded as 1 or 2 are recoded as 1, those who originally responded as 3 or 4 are recoded as 2, and those who originally responded as 5 or 6 are recoded as 3.

***Environmental Protection*** (`impenv`): This is an ordinal variable measuring respondents' opinions about that it is important to care for nature and environment. This variable is originally coded from a scale of 1 (very much like me) to 6 (not like me at all). We further recode this variable as a three-point scale: respondents who originally responded as 1 or 2 are recoded as 1, those who originally responded as 3 or 4 are recoded as 2, and those who originally responded as 5 or 6 are recoded as 3.

***Tradition*** (`imptrad`): This is an ordinal variable measuring respondents' opinions about that it is important to follow traditions and costumes. This variable is originally coded from a scale of 1 (very much like me) to 6 (not like me at all). We further recode this variable as a three-point scale: respondents who originally responded as 1 or 2 are recoded as 1, those who originally responded as 3 or 4 are recoded as 2, and those who originally responded as 5 or 6 are recoded as 3.
