# Supplementary material for: Contrastive multiple correspondence analysis (cMCA): Using contrastive learning to identify latent subgroups in political parties
Source: PLoS One. 2023 Jul 10;18(7):e0287180. doi: 10.1371/journal.pone.0287180 (PMC10332614; doi:10.1371/journal.pone.0287180)
Supplement: Attachment — Submitted filename: review.pdf [file pone.0287180.s007.pdf]

Review: “Contrastive Multiple Correspondence Analysis (cMCA): Using Contrastive Learning to Identify Latent Subgroups in Political Parties”

November 30, 2022

The authors propose a contrastive learning method developed expressly for categorical data, contrastive Multiple Correspondence Analysis (cMCA). cMCA is applied to two survey datasets, and its results are discussed.

Major Comments

- I’m a little puzzled by the limitations of traditional scaling methods outlined on pages 2 and 3. If researchers are seeking latent factors within sub-populations, like Democrats in the House of Representatives, instead of across a population, like the entirety of the House of Representatives, why not apply these scaling methods directly to the sub-population of interest? This should be addressed in the motivating example. Applying vanilla MCA to the subgroups in the analyses of Section 3, followed by a comparison of MCA’s latent factors to those produced by cMCA would also serve as an important demonstration of the proposed methods superiority over traditional methods.

- It’s stated that cMCA “objectively” and “agnostically” identifies subgroups in the last paragraph of page 4. Can you explain what is meant by these adverbs?

- Is it appropriate to claim that cMCA is objective given that analysts are encouraged to manually select the value of α that produces the most “meaningful” latent space? Wouldn’t this approach bring about spurious findings?

- Can you provide more information on the CES 2020 and ESS 2018 surveys for readers who might be unfamiliar with these data sources? In particular, how many observations and features are contained in each? How many respondents and questions were removed during preprocessing?

- I feel that the methodological contributions of this work are overstated. Just as MCA is PCA applied to data whose categorical variables are transformed into dummy variables, cMCA is cPCA applied to one-hot encoded data. cMCA is a less general form of cPCA that is limited to categorical data. I’d instead focus on the following: This work demonstrates that contrastive methods, like cPCA, might provide novel insights overlooked by traditional methods when analyzing categorical survey data.

Minor Comments

- Page 3, second paragraph: "Variation that is statistically small across groups may be significantly large within groups, as seen in substantive applications in political science." I don't quite understand this sentence. What does it mean to for variation to be "statistically small"?
- Page 4, second paragraph: I believe that there's a typo in "(due to variable-types cannot be analyzed)".
- $\mathbf{L}$ is undefined when one of the K' leading eigenvalues is negative. Perhaps this should be mentioned in the paragraph following Equation (11).
