# Supplementary material for: Contrastive multiple correspondence analysis (cMCA): Using contrastive learning to identify latent subgroups in political parties
Source: PLoS One. 2023 Jul 10;18(7):e0287180. doi: 10.1371/journal.pone.0287180 (PMC10332614; doi:10.1371/journal.pone.0287180)
Supplement: Attachment — Submitted filename: cMCA_Response_Letter.pdf [file pone.0287180.s008.pdf]

Response Memo: “Contrastive Multiple Correspondence Analysis (cMCA): Using Contrastive Learning to Identify Latent Subgroups in Political Parties”

Dear Editor and Reviewers:

We appreciate the chance to submit a revised version of “Contrastive Multiple Correspondence Analysis (cMCA): Using Contrastive Learning to Identify Latent Subgroups in Political Parties” to *PLOS One*. Our revision responds to the concerns raised by all of the reviewers and we believe that the revised version has a stronger, and clearer, contribution to the literature. We are grateful to everyone involved for taking the time to give such professional and helpful feedback. Below we provide our response to each of the points raised. For the convenience of the review, all the revisions of the manuscript appear in blue color text on the revised manuscript so specific revisions can be more easily conveyed.

Reviewer 1

a This is an excellent and interesting paper that introduces an analysis technique for identifying subgroup latent patterns. I particularly value the clarity by which the methods and calculations are explained, which is comprehensive and easy to follow. The choice of context to apply the analysis also showcases its usefulness.

Response: We thank Reviewer 1 for their favorable evaluation and recognition of this project’s contribution to the literature.

b On page 8 you say the matrices must have the same dimensions, which I take to mean that the two groups for comparison must be the same size. Could you please a) clarify this and b) discuss whether the analysis can be performed on groups of different sizes. It seems that the requirement for symmetrical groups limits the application of this technique, or forces the researcher to make decisions about which observations to include in the groups in order to balance them. If this is the case, could you explain how to tackle asymmetrical groups should there be no way round it (i.e with some pre analysis).

Response: As shown in Equation 1, the procedure of “contrasting” in cMCA is a subtraction of two Burt matrices ($\mathbf{B}_T$ and $\mathbf{B}_B$), and thus, these two matrices must have the same matrix size (rows and columns). Nevertheless, this does not mean two groups ($\mathbf{X}_T$ and $\mathbf{X}_B$) have to have the same sample size. As long as the two datasets have an identical set of

variables, their Burt matrices are guaranteed to have the same dimensions, i.e., the same numbers of columns and rows. The contrastive procedure only compares the variance-covariance of variables captured in the Burt matrices but does not directly compare the original data. To clarify this point, we first replaced "matrix difference" with "matrix subtraction" on page 8. In addition, we have added an explanation in the footnote at the end of the paragraph on page 8 as follows:

Note that this does not mean that the original datasets of two groups must have the same sample size, as expressed as $\mathbf{X}_T \in \mathbb{R}^{n\times d}$ and $\mathbf{X}_B \in \mathbb{R}^{m\times d}$ (on the other hand, the original datasets of two groups must have the same set of d variables). Both cPCA and cMCA only compare the variance-covariance or Burt matrices of two original datasets. When two datasets have the same set of variables, their variance-covariance/Burt matrices are guaranteed to have the same dimensions.

c Relatedly, your first step in preparing the data for application of the method involves removing missing cases. Could you please discuss what the consequences are (if any) for removing missingness.

Response: This feedback points to something important but missed in the discussion. To elaborate on the potential consequences of the listwise deletion, we have added a footnote on page 14 as follows:

Due to the focus of this paper being the usage of cMCA, we assume that the missing data is generated at random in our analyses; however, note that when the data is not missing at random, listwise deletion could cause analytical results to be inefficient and biased (King et al. 2001).

d A minor point but it is not immediately clear whether the CES and ESS are citizen surveys unless you are familiar with the data because in the earlier parts of the paper you talk about analysing Congress members. I recommend a sentence just clarifying this.

Response: We thank Reviewer 1's comment. Indeed, the surveys utilized in this project may not be universally familiar to everyone, especially those who are not from the subfield of political science. Following the instruction, on page 4, we have added sentences to briefly introduce these two surveys as follows:

To demonstrate the utility of cMCA, we analyze two citizen surveys, the 2020 Cooperative Election Survey (CES 2020), which investigates general political attitudes, various demographic factors, assessment of roll call voting choices, political information, and vote intentions of American voters; and the U.K. module of the 2018 European Social Survey (ESS-UK 2018),

which is a biennial cross-national survey of attitudes and behaviors across member countries.

e It would be useful to see in the main text a table or diagram of what is in the analysis that produces Figures 1 and 3, i.e. the substantive variables not just the variable codes as in the appendix. I would also recommend an extra sentence for each of the interpretations of these visualisations to make clear to all types of readers what is seen there, for instance "We can see this ideological polarisation because..."

Response: We understand that it is not easy for readers to engage in the analytical results when they are only provided with variable codes instead of their substantive names. Although we originally prepared an online appendix reporting every variable's code name and their assigned substantive names, mistakenly, we did not attach it to our first submission. Due to the limited space, we have chosen to adopt our original strategy—communicating with readers regarding variables' code names and substantive names through an online appendix—and have added as supporting information (please refer to S5).

f If the main strength of this analysis is that it shows patterns/difference in variation, I wonder if you could more clearly describe or quantify this. For instance, 'Democrats are twice as similar to Republicans on the Supreme Court dimension as they are on Trump approval' or some kind of interpretation in the text like this.

Response: We thank Reviewer 1's comment on the potential usage of cMCA. However, given that the two (contrastive) subspaces are constructed by different feature/variable combinations, according to our knowledge, such mentioned comparison (i.e., "Democrats are twice as similar to Republicans on the Supreme Court dimension as they are on Trump approval") involves in the technique called **bridging** and requires at least one "anchor" (i.e., the one who happens to be in both groups). Thus, when only relying on the current cMCA, we think such a comparison is difficult. Nevertheless, we believe that how to bridge two separate contrastive spaces is a valuable topic for future research.

g Finally, another minor point but in the UK analysis (Figures 4 and 5) I would recommend making the background data point larger so it can clearly be seen. I am unsure why it is a large group in the US analysis but looks small in the UK – perhaps this is due to N and this could be reported?

Response: We have made the background data point larger in Figures 4 and 5. As seen in Equations 6 and 7, when a large alpha is selected by the automatic selection (e.g., close to 1000 in Figures 4 and 5), the

variance/scatteredness difference of target and background data points in a latent space will be large as well. This is the reason why background data points have located in a very small region in Figures 4 and 5 when compared with Figure 2.

Reviewer 2

a I'm a little puzzled by the limitations of traditional scaling methods outlined on pages 2 and 3. If researchers are seeking latent factors within sub-populations, like Democrats in the House of Representatives, instead of across a population, like the entirety of the House of Representatives, why not apply these scaling methods directly to the sub-population of interest? This should be addressed in the motivating example. Applying vanilla MCA to the subgroups in the analyses of Section 3, followed by a comparison of MCA's latent factors to those produced by cMCA would also serve as an important demonstration of the proposed methods superiority over traditional methods.

Response: Based on this comment, we have revised the third paragraph of **Introduction** to qualitatively explain why applying vanilla MCA to one party may not generate a similar result to the result of cMCA:

Variation that is overlooked *across* groups may be significant *within* groups, as seen in substantive applications in political science. Indeed, traditional scaling methods only uncover certain intraparty divisions that are related to the "mainstream" pattern (e.g., when applying scaling methods only to Democrats or Republicans, the explored subgroups are usually consistent with ideological differences).

Furthermore, we have applied vanilla MCA to Democrats, Republicans, Labours, and Conservatives separately and provide figures of category loadings of PC1 in Appendix F, as comparisons to the results of cMCA. As one can see, the comparisons of results of cMCA and MCA on single groups demonstrate that such intraparty division may be covered by the main division across groups, as we argue in the main text. The results of CES 2020 demonstrate that the structure of PC1 generated by MCA is similar to the one generated by cMCA but with more noise. For Democrats, although the structure of PC1 generated by MCA is similar to the one generated by cMCA, the controversies over the supreme court nomination are no more the most influential features in the MCA result. Similar to Republicans, the controversies over Trump are also no more the most influential features in the MCA result. Furthermore, the results of ESS-UK 2018 show that the structure of PC1 generated by MCA is totally different from the one generated by cMCA—the EU issues dominate PC1 of both Labours' and Conservatives' MCA results and the left-right ideology is the single most significant feature of PC1 of both their cMCA

results. To demonstrate this point, we have added a footnote at the end of the second paragraph of **Discussion** on page 27 as follows:

To demonstrate this point, we apply MCA to only Democrats, Republicans, Labours, and Conservatives and provide category loadings of PC1 of each result in Appendix F as comparisons to the cMCA results. As one can see, applying MCA to each of the two single groups separately does not generate the same results as applying cMCA to the two groups together. The PCs generated by MCA are either totally different from those generated by cMCA or still similar to them but with more noise.

b It's stated that cMCA "objectively" and "agnostically" identifies subgroups in the last paragraph of page 4. Can you explain what is meant by these adverbs?

Response: Based on Reviewer 2's confusion of the meaning of the selected terms (i.e., objectively and agnostically), we have removed "agnostically" in the draft and have explained what we mean by "objectively" in the footnote on page 5 as follows:

By objectivity, we mean that instead of picking variables/attributes based on researchers' own (subjective) prior beliefs regarding the cause of intra polarization, cMCA (or contrastive learning in general) explores these attributes by recognized statistical properties/criteria, e.g., variables which cause the largest variance to the distribution.

c Is it appropriate to claim that cMCA is objective given that analysts are encouraged to manually select the value of α that produces the most "meaningful" latent space? Wouldn't this approach bring about spurious findings?

Response: By "meaningful" latent space, we mean the space which demonstrates "clear patterns of data division" or "clear patterns of data clustering." To prevent such potential confusion, we have first removed "meaningful" from three separate sentences. At the end of the first paragraph on page 5, we have revised the original sentence "cMCA's results provide important information for researchers to better understand and make meaningful distinctions between underlying subgroups in their data" to "cMCA's results provide important information for researchers to better understand and make distinctions between underlying subgroups in their data"; at the end of the last paragraph on page 27, we have revised the original sentence "...either with manual- or auto-selection, to derive latent dimensions or traits which could subgroup data in meaningful ways" to "...either with manual- or auto-selection, to derive latent dimensions or traits which could subgroup data"; finally, in the next paragraph on page 28, we have revised the original sentence "...it is difficult

for researchers to effectively apply CSA and sMCA to objectively explore meaningful subgroups" to "...it is difficult for researchers to effectively apply CSA and sMCA to objectively explore subgroups." In addition, at the end of the first paragraph of the subsection "Selection of the contrast parameter," we replace "meaningful latent space" by "clear patterns of data division/clustering" as follows:

Thus, researchers can manually explore different α values to seek latent spaces that demonstrate clear patterns of data division/clustering for them.

d Can you provide more information on the CES 2020 and ESS 2018 surveys for readers who might be unfamiliar with these data sources? In particular, how many observations and features are contained in each? How many respondents and questions were removed during preprocessing?

Response: The similar comment is provided by Reviewer 1 (Reviewer 1-d). In addition, we follow Reviewer 2's suggestion to describe the number of variables included and the change of sample size for both surveys. In the first paragraph of the subsection **Case one: CES 2020—the hidden conflicts regarding Trump and related issues** (on page 15), we have revised and added two sentences as follows:

- For this analysis, we focus on the pre-election wave and select 69 variables all related to national issues, including job approval, roll call votes, and salient policies and issues (e.g., abortion and gun control).
- After deletion, the sample size reduces from 7,700 respondents to 5,616.

Furthermore, in the first paragraph of the subsection **Case two: ESS-UK 2018—the Brexit cleavage in the Labour and Conservative parties** (on page 20), we have revised and added two sentences as follows:

We first manually select 23 variables which are generally related to political/social issues and recode this data through the aforementioned procedure. For simplicity, we only include three partisans: the Conservative, Labour, and UK Independence Party (UKIP). After deletion the sample size decreases from 946 respondents to 777.

e I feel that the methodological contributions of this work are overstated. Just as MCA is PCA applied to data whose categorical variables are transformed into dummy variables, cMCA is cPCA applied to one-hot encoded data. cMCA is a less general form of cPCA that is limited to categorical data. I'd instead focus on the following: This work demonstrates that contrastive methods, like cPCA, might provide novel insights overlooked

by traditional methods when analyzing categorical survey data.

Response: We thank Reviewer 2's suggestion and at the end of the second paragraph of **Discussion** (on page 27), we have rewritten the suggested words and replaced the original sentence as follows:

this work demonstrates that cMCA, or contrastive methods in general, might provide novel insights overlooked by traditional methods when analyzing categorical survey data.

f Page 3, second paragraph: "Variation that is statistically small across groups may be significantly large within groups, as seen in substantive applications in political science." I don't quite understand this sentence. What does it mean to for variation to be "statistically small"?

Response: We agree that the term "statistically small" is confusing to the readers. What we try to describe is that sometimes some intraparty variation may be hidden by the "major variation" across groups so as to demonstrate the usage of cMCA. To avoid such confusion, we have revised the beginning of the third paragraph of **Introduction** as follows:

Variation that is overlooked *across* groups may be significant *within* groups, as seen in substantive applications in political science.

Reviewer 3

a This is a well-written and highly innovative paper that combines two scaling methods and demonstrates their utility with two substantive examples. One of these scaling approaches (contrastive learning) is novel and stems largely from Abid et al.'s 2018 paper in Nature Communications. The other (Multiple Correspondence Analysis, or MCA) is a more established scaling technique, though it still underutilized and underappreciated in much of the social sciences, especially political science.

By combining the two, the paper provides an immensely valuable tool for social scientists looking to uncover heterogeneity in attitude structures. While much work has been done on developing methods for identifying heterogeneity in the context of treatment effects from experimental (and occasionally observational) data, very little effort has been put into extending these tools in the field of scaling methodology. Moreover, many of us in the scaling community have long felt that MCA should be more widely applied to political science problems, and I am happy to see this paper put it to good use here. Finally, I think the paper does a terrific job of walking the reader through these different components, explaining their mechanisms, and motivating their usage. The two substantive examples

are relevant and effectively demonstrate the value of the contrastive MCA (cMCA) method in recovering richer sources of attitudinal heterogeneity among voters.

Response: We thank Reviewer 3 for their kind assessment and acknowledgment of this project's contribution to the literature.

b The first two paragraphs of the Introduction are a bit clunky. They divide scaling methods into two rough groups: "optimal scoring" (PCA, CA/MCA, and factor analysis) and "spatial voting" (NOMINATE, IRT, Optimal Classification, and others). This strikes me as an unconventional organization, especially because IRT models are a cumulative (vs. unfolding) scaling method that have been applied to measure and test spatial voting even though they are not a proximity model (as are unfolding methods like NOMINATE and OC). Admittedly many of these differences are technical/semantic. But I think that if the paper is going to use this framework, it should better explain the difference between the optimal scoring and spatial voting classes of methods. Jacoby's (1991) Sage green book on Data Theory and Dimensional Analysis might provide a nice source.

Response: We thank Reviewer 3 for pointing out this issue. We admit that these two "categories" are not general enough. We have followed Reviewer 3's suggestion to categorize scaling methods under two common categories: accumulative and unfolding models. In addition, we have also introduced other approaches that belong to none of the two, including PCA, MCA, etc. The beginning paragraph of Introduction is revised as follows:

This has been accomplished by utilizing one of two general ideas: accumulative and unfolding models (Armstrong et al. 2014; Duck-Mayr and Montgomery 2023; William G Jacoby 1999). The widely utilized accumulative model is the classic item response theory (IRT) model, which re-scales the data by a arranging series of questions in order of their level of discrimination regarding the measurement of certain latent traits (Armstrong et al. 2014). On the contrary, unfolding models (e.g., the NOMINATE class of models and Optimal Classification (OC)) assume that each individual has an ideal point and this individual's preference is modeled by a single-peaked function, which translates proximity information into distances in a latent space (Duck-Mayr and Montgomery 2023; William G. Jacoby 1991). While these methods have been most frequently applied to roll-call votes in the United States Congress or other political bodies, scaling methods that do not belong to the above categories such as PCA, MCA, black-box scaling, ordered OC (OOC), and ordinal IRT (OIRT) have been increasingly used to analyze a range of new data, especially surveys, to explore underlying political patterns among citizens (Blasius and Thiessen 2001; Hare, Liu, and Lupton 2018; Imai, Lo, and Olmsted

2016; Monroe, Colaresi, and Quinn 2008; Poole 1998; Wilkerson and Casas 2017).

c I think Poole's "optimal classification" method should be capitalized.

Response: We thank Reviewer 3's comment and the term Optimal Classification is capitalized in the draft.

d In the second paragraph of the Introduction, I'm not sure how accurate it is to list Coombs (1964) as the origin of the consistent finding of a left-right ideological organizing dimension. Certainly, Coombs' work was hugely influential, but Coombs was a psychometrician and did very little work directly in political science. I think it would be more appropriate to cite the work that provided the foundation of what would become known as the "basic space theory of ideology" (Hinich and Munger 1994). The specific literature is varied, but some notable work includes Weisburg and Rusk (1970, "Dimensions of Candidate Evaluation"), Cahoon et al. (1978, "A Statistical Multidimensional Scaling Method Based on the Spatial Theory of Voting"), Rabinowitz (1973, "Spatial Models of Electoral Choice"), and of course Downs (1957).

Response: We thank Reviewer 3's comment. Indeed, the whole idea of ideal point estimation is based on spatial models. The beginning of the second paragraph of Introduction is revised as follows:

Ever since Downs (1957), the spatial modelers have theoretically and empirically posited the existence of a single-dimensional ideological space (e.g., Cahoon, Hinich, and Ordeshook 1978; Hinich and Munger 1992; Rabinowitz 1973), and the latent left-right dimension of ideology is often recovered as an important principal component (PC) in political data (e.g., Coombs 1964; Poole and Rosenthal 1991; Poole 2000).

e I think "U.S. Congresspersons" should be "U.S. Members of Congress (MCs)". This is a more common term.

Response: We thank Reviewer 3's suggestion and have revised "U.S. Congresspersons" as "U.S. Members of Congress (MCs)" in the draft.

f I think the third paragraph of the Introduction could simply mention that traditional scaling methods only uncover certain kinds of intraparty divisions (e.g., the "Squad" or the Tea Party caucus) that are related to the general left-right dimension.

Response: We thank Reviewer 3's suggestion and have simplified the beginning of the third paragraph of Introduction as follows:

Response: Variation that is overlooked *across* groups may be significant *within* groups, as seen in substantive applications in political science. Indeed, traditional scaling methods only uncover certain kinds of intraparty divisions that are related to the "mainstream" pattern (e.g., when applying scaling methods only to Democrats or Republicans, the explored subgroups are usually consistent with ideological differences).

g On p. 4, I would change the first sentence in the final paragraph to "and the UK module of the 2018 European Social Survey (ESS-UK 2018)".

Response: We thank Reviewer 3's suggestion and have revised the abbreviation of the "UK module of the 2018 European Social Survey" as ESS-UK 2018 across the whole draft.

h At some point (perhaps in Section 2.1 on p. 6), I think the paper should mention that MCA is a valuable tool for representing categorical variation in data but it has not been widely utilized in political science. However, there are exceptions such as Bonica (2014, "Mapping the Ideological Marketplace"); Gibson and Hare (2016, "Moral Epistemology and Ideological Conflict"); Blasius and Thiessen (2001, "Methodological Artifacts in Measures of Political Efficacy and Trust"), and perhaps others.

Responses: We appreciate Reviewer 3's comment. This way would make the contribution of this project stronger. At the end of the first paragraph of the second section, we have added a footnote as follows:

MCA is a valuable tool for exploring principle components of categorical data; however, it has not been widely utilized in political science (with several exceptions, such as Bonica (2014), Blasius and Thiessen (2001), and Gibson and Hare (2016)).

i Perhaps I'm being dense, but is there a parallel between the contrast parameter α and the row/column scaling weight in biplots? If so, the authors might consider mentioning this parallel as a familiar point of comparison for the reader.

Response: The function of α is for demonstration of the division of data. Therefore, it does not involve in the procedure of biplot. Once the "best" α is obtained, researchers use the vectors of principle directions to create the latent space and thus the biplot.
