# Supplementary material for: Contrastive multiple correspondence analysis (cMCA): Using contrastive learning to identify latent subgroups in political parties
Source: PLoS One. 2023 Jul 10;18(7):e0287180. doi: 10.1371/journal.pone.0287180 (PMC10332614; doi:10.1371/journal.pone.0287180)
Supplement: Attachment — Submitted filename: review-2.pdf [file pone.0287180.s009.pdf]

Review 2: "Contrastive Multiple Correspondence Analysis (cMCA): Using Contrastive Learning to Identify Latent Subgroups in Political Parties"

April 21, 2023

I'd like to thank the authors for attempting to address most of my comments in this revision. It's exciting to see contrastive methods being developed and used in new disciplines. While the authors have clearly put in great effort to responding the reviewers comments and suggestions, I believe that there are some outstanding issues relating the presentation of existing methods and of the proposed methodology that need to be addressed. Because PLOS One's audience is made up of scientists from a diversity of fields, statistical methodology must be presented as generally as possible. While I'm certain that the authors are presenting all methods in a fashion consistent with other works in their substantive areas, I hope that my suggestions will make this manuscript more accessible to a PLOS One's readership.

1. "However, given that these scaling methods analyze complete data and only derive general patterns, they do not provide effective analyses for cases where researchers' interests are on latent patterns within individual groups (e.g., citizens supporting different political parties) rather than those seen across groups": As I stated in my previous review, this statement is inaccurate. Scaling methods like PCA can be applied to the individual subgroups—assuming these groups are known—to learn the latent patterns within these subgroups. You might find that the leading latent patterns in two subgroups are qualitatively similar to the latent patterns obtained by applying the scaling method to the two subgroups simultaneously, but this is not true in generality. When this is the case, however, contrastive methods may help tease out the latent factors that are unique to a subgroup. I think your motivation for using contrastive methods should reflect this line of reasoning.

2. Thank you for providing the category loadings in Figures 30 and 31. While these plots are insightful, a more digestible comparison of MCA to cMCA would be to simply recreate the Figures 2, 4, and 5 using MCA. That is, for example, Figure 2c could be recreated by applying MCA to the `Dem` dataset, and color the observations in the low-dimensional embedding by attitude towards the Supreme Court judge nominations. If the resulting plot is qualitative different from Figure 2c, then you have demonstrated that cMCA more recovers patterns that MCA can't.

3. On the distinction between PCA and MCA, and cPCA and cMCA: As alluded to in my previous review, it's incorrect to state that (contrastive) PCA is only applicable to continuous data. This claim at odds with the broader literature on PCA. For exploratory purposes, (contrastive) PCA does not have distributional assumptions and can be used on all kinds of data [Jolliffe and Cadima, 2016]. For example, consider the application of PCA to categorical genotype data [Gauch et al., 2019]. In general, practitioners do not omit categorical

variables from datasets prior to performing PCA, they instead one-hot encode these variables and continue the analysis as planed. Instead of claiming that PCA is inapplicable in the categorical data setting, as is done in introduction and in the "Multiple correspondence analysis (MCA)—an alternative to PCA" section, it would be more honest to state that PCA can be applied to categorical data with a simple transformation of the data, and that, in some fields like political science, this procedure is commonly referred to as MCA.

4. Technical detail: The optimization problem in Equation 1 is must be defined with greater care; $\mathbf{u}$ must be a vector with a bounded Euclidean norm. Otherwise, the solution is not unique. As such, $\mathbf{u}$ is typically restricted to unit vectors. Instead of specifying that $\|u\| = 1$ on line 175, it should be specified earlier on line 164.

5. Nomenclature issue: On line 185-186, the eigenvectors obtained by performing cMCA on the $(\mathbf{B}_T - \alpha\mathbf{B}_B)$ are referred to as contrastive principal components. This is at odds with much of the (contrastive) PCA literature. Eigenvectors are generally referred to as loadings, and the vectors obtained by the matrix multiplication of the original data and the eigenvectors (loadings) are named the (contrastive) principal components [Jolliffe and Cadima, 2016]. This is a relatively common mistake—even Abid et al. [2018] made it when describing cPCA.

6. Equation 11: I mentioned this in my previous review, but it wasn't addressed: While the eigenvalues produced by PCA/MCA will always be positive, that is not the case with cPCA/cMCA. When $\boldsymbol{\lambda}$ contains negative elements, $\mathbf{L}$ is undefined. How should users account for this?

7. Including background data in cMCA plots: While including the background dataset in the low-dimensional cMCA embeddings is a helpful diagnostic to make sure that the contrastive procedure is working as expected, it makes it more difficult to appreciate the patterns unique to the target dataset due to over plotting. It's uncommon to include the background datasets in contrastive low dimensional embeddings since they're expected to cluster tightly around a single point anyways. Please consider removing the background data from Figures 2c, 2d, 4c-4f, 5c, and 5d. This will also facilitate the comparison with MCA that I mentioned in point 2 above.

Again, this is exciting work that merits publication. However, I think that the presentation of statistical methodology must be modified so that it can be fully appreciated by a wide audience.

References

A. Abid, M. J. Zhang, V. K. Bagaria, and J. Zou. Exploring patterns enriched in a dataset with contrastive principal component analysis. *Nature Communications*, 9(1):2134, 2018.

H. G. Gauch, Jr., S. Qian, H.-P. Piepho, L. Zhou, and R. Chen. Consequences of pca graphs, snp codings, and pca variants for elucidating population structure. *PLOS ONE*, 14(6):1–26, 06 2019.

I. T. Jolliffe and J. Cadima. Principal component analysis: a review and recent developments. *Philosophical Transactions of the Royal Society A: Mathematical, Physical and Engineering Sciences*, 374(2065):20150202, 2016.
