# Supplementary material for: Contrastive multiple correspondence analysis (cMCA): Using contrastive learning to identify latent subgroups in political parties
Source: PLoS One. 2023 Jul 10;18(7):e0287180. doi: 10.1371/journal.pone.0287180 (PMC10332614; doi:10.1371/journal.pone.0287180)
Supplement: Attachment — Submitted filename: cMCA_Response_Leter_2.pdf [file pone.0287180.s010.pdf]

Response Memo II: "Contrastive Multiple Correspondence Analysis (cMCA): Using Contrastive Learning to Identify Latent Subgroups in Political Parties"

Dear Editor and Reviewers:

We appreciate the second chance to submit a revised version of "Contrastive Multiple Correspondence Analysis (cMCA): Using Contrastive Learning to Identify Latent Subgroups in Political Parties" to *PLOS One*. Our revision responds to the concerns raised by Reviewer 2 and the editor. As Reviewer 2 mentioned in the comment, because "PLOS One's audience is made up of scientists from a diversity of fields, statistical methodology must be presented as generally as possible," and we believe that the revised version has a stronger, and clearer, contribution to the literature. Below we provide our response to each of the points raised. For the convenience of the review, all the revisions of the manuscript appear in blue color text on the revised manuscript so specific revisions can be more easily conveyed.

Reviewer 2

a "However, given that these scaling methods analyze complete data and only derive general patterns, they do not provide effective analyses for cases where researchers' interests are on latent patterns within individual groups (e.g., citizens supporting different political parties) rather than those seen across groups": As I stated in my previous review, this statement is inaccurate. Scaling methods like PCA can be applied to the individual subgroups—assuming these groups are known—to learn the latent patterns within these subgroups. You might find that the leading latent patterns in two subgroups are qualitatively similar to the latent patterns obtained by applying the scaling method to the two subgroups simultaneously, but this is not true in generality. When this is the case, however, contrastive methods may help tease out the latent factors that are unique to a subgroup. I think your motivation for using contrastive methods should reflect this line of reasoning.

Response: We thank Reviewer 2's comment and apologize for not dealing with this part properly. We agree with this comment that the issue we identify here is not always the case. In this current version, we have revised the quoted paragraph above as follows:

However, as in the aforementioned ideology PC, the general structure and related patterns may not be particularly interesting or important to researchers. Rather, using alternatives, researchers may be interested in

exploring *specific structure and patterns within each of groups* (e.g., citizens supporting different political parties).

Furthermore, we have also revised the tone at the end of the second paragraph of Introduction as follows:

It is important to note that we by no means consider existing models to be problematic or inferior—we expect an alternative approach that focuses on variation specific within each group to be especially fruitful in cases where subgroups may lurk in the data and be overlooked by ordinary approaches.

b Thank you for providing the category loadings in Figures 30 and 31. While these plots are insightful, a more digestible comparison of MCA to cMCA would be to simply recreate the Figures 2, 4, and 5 using MCA. That is, for example, Figure 2c could be recreated by applying MCA to the Dem dataset, and color the observations in the low-dimensional embedding by attitude towards the Supreme Court judge nominations. If the resulting plot is qualitative different from Figure 2c, then you have demonstrated that cMCA more recovers patterns that MCA can't.

Response: We agree with Reviewer 2's comment that the current way of comparing the results of MCA and cMCA is a little clunky, and might be hard to digest for readers. Nevertheless, due to the characteristic of the selected data, we would like to ask for agreement to include both our original way of demonstrating the differences between the two approaches and the way suggested by the reviewer given that the suggested way may not work well for certain cases. To see this, taking the Democrats as an example, although the linear combinations of principal components are different between the MCA and the cMCA results, the feeling of Democrats toward the nomination of Amy Coney Barrett is still the most influential variable for both cases. Thus, if we color code the Democrats by their opinions on Amy Coney Barrett's confirmation in both cases, we will not be able to tell the differences well and this, in turn, may reduce the utility of cMCA. The same issue happens when we analyze Republicans only. Overall, to respond to this suggestion, we have decided to keep the original figures in S4, add new figures of color-coded MCA results in S4, and revise the explanatory footnote in the second paragraph of Discussion as follows:

To demonstrate this point, we apply MCA to only Democrats, Republicans, Labours, and Conservatives and provide category loadings of PC1 of each result in S4 Appendix as comparisons to the cMCA results in S3 Appendix. As one can see, for the case of Democrats and Republicans, given that the linear combination or the structure of PCs derived by different approaches are not the same (i.e., the sets of top-5 most influential

variables derived by different approaches are not identical), this demonstrates that applying MCA to each of the two single groups separately does not generate the same results as applying cMCA to the two groups together. Furthermore, for the cases of Labours and Conservative, one can tell the differences between MCA and cMCA by simply eye-testing their color-coded results. Through the comparison, we find that the structure of PCs generated by MCA is either totally different from those generated by cMCA (the Conservatives and Labours) or still similar to them but with more noise (the Democrats and Republicans).

c On the distinction between PCA and MCA, and cPCA and cMCA: As alluded to in my previous review, it's incorrect to state that (contrastive) PCA is only applicable to continuous data. This claim at odds with the broader literature on PCA. For exploratory purposes, (contrastive) PCA does not have distributional assumptions and can be used on all kinds of data [Jolliffe and Cadima, 2016]. For example, consider the application of PCA to categorical genotype data [Gauch et al., 2019]. In general, practitioners do not omit categorical variables from datasets prior to performing PCA, they instead one-hot encode these variables and continue the analysis as planed. Instead of claiming that PCA is inapplicable in the categorical data setting, as is done in introduction and in the "Multiple correspondence analysis (MCA)—an alternative to PCA" section, it would be more honest to state that PCA can be applied to categorical data with a simple transformation of the data, and that, in some fields like political science, this procedure is commonly referred to as MCA.

Response: Following this suggestion, we have revised the first paragraph of the subsection "Multiple correspondence analysis (MCA)—an alternative to PCA" as follows:

Although PCA has been widely used across disciplines for scaling,...That is, as PCA assumes the relationships between variables are *linear* (Linting et al. 2007), PCA does not effectively analyze categorical data (Gauch Jr et al. 2019)—including binary data, nominal data, and ordinal data, where the parallel slopes assumption does not often hold (Long 1997). One of the standard practices to resolve this issue is to create dummy variables for all categorical data, then standardize the whole data, and finally apply PCA to this new dataset—this procedure is commonly recognized as a variant of PCA for categorical data and referred to as *multiple correspondence analysis* (MCA) by some scholars (Greenacre 2017).

d Technical detail: The optimization problem in Equation 1 is must be defined with greater care; $\mathbf{u}$ must be a vector with a bounded Euclidean norm. Otherwise, the solution is not unique. As such, $\mathbf{u}$ is typically restricted to unit vectors. Instead of specifying that $||u|| = 1$ on line 175, it

should be specified earlier on line 164.

Response: We have revised the first and second paragraphs based on Reviewer 2's comment as follows (mainly in the first):

Let $\mathbf{u}$ be any K-dimensional vector. Similar to cPCA, we can derive the variances of the target and background groups along $\mathbf{u}$ with $\sigma_T^2(\mathbf{u}) \stackrel{\text{def}}{=} \mathbf{u}^\top \mathbf{B}_T \mathbf{u}$ and $\sigma_B^2(\mathbf{u}) \stackrel{\text{def}}{=} \mathbf{u}^\top \mathbf{B}_B \mathbf{u}$. In addition, since we want to find a "direction" (i.e., we are not interested in the length of $\mathbf{u}$), we impose a constraint that sets the derived direction to be a *unit vector*, i.e., $\|\mathbf{u}\| = 1$. Let Σ denote $(\mathbf{B}_T - \alpha \mathbf{B}_B)$. To find the direction, $\mathbf{u}^*$, ...

e Nomenclature issue: On line 185-186, the eigenvectors obtained by performing cMCA on the $(\mathbf{B}_T - \alpha \mathbf{B}_B)$ are referred to as contrastive principal components. This is at odds with much of the (contrastive) PCA literature. Eigenvectors are generally referred to as loadings, and the vectors obtained by the matrix multiplication of the original data and the eigenvectors (loadings) are named the (contrastive) principal components [Jolliffe and Cadima, 2016]. This is a relatively common mistake—even Abid et al. [2018] made it when describing cPCA.

Response: We appreciate Reviewer 2 for pointing out this nomenclature issue. We have revised the parts referring to cPCs. Also, we introduced cPCs when we describe data-points coordinates as follows:

We call this new space's axes (i.e., linear combinations of the original categories and the eigenvectors) *contrastive principal components* (cPCs).[10]

(Footnote 10) Although the eigenvectors, $\mathbf{U}$, are sometimes called cPCs in existing literature (e.g., [12, 14]), it is inaccurate according to Jolliffe and Cadima [37].

f Equation 11: I mentioned this in my previous review, but it wasn't addressed: While the eigenvalues produced by PCA/MCA will always be positive, that is not the case with cPCA/cMCA. When λ contains negative elements, $\mathbf{L}$ is undefined. How should users account for this?

Response: We thank Reviewer 2 for pointing out this issue. We did respond to this comment and apologize for forgetting to include our responses in the response letter. To address this issue, we added a footnote on line 255 as follows:

For negative eigenvalues, $\mathbf{L}$ is undefined. However, in practice, we only take a few top eigenvalues for analyses, and usually, we can expect that they are positive.

g Including background data in cMCA plots: While including the background dataset in the low-dimensional cMCA embeddings is a helpful diagnostic to make sure that the contrastive procedure is working as expected, it makes it more difficult to appreciate the patterns unique to the target dataset due to over plotting. It's uncommon to include the background datasets in contrastive low dimensional embeddings since they're expected to cluster tightly around a single point anyways. Please consider removing the background data from Figures 2c, 2d, 4c-4f, 5c, and 5d. This will also facilitate the comparison with MCA that I mentioned in point 2 above.

Response: We agree that projecting a background dataset increases the number of points in the plot and may cause overplotting issues. However, as in this review comment and our discussion on Page 7, plotting the background dataset is helpful to diagnose whether or not the embedding has the situation of high target variance and low background variance. Also, the background datasets in our plots in Application section do not strongly disturb the recognition of the target datasets' patterns. Thus, we would like to keep the current plots. We also want to note that, with our open-source library of cMCA, users can easily switch this option.
